# Assessing the Genetics Content in the Next Generation Science Standards

**DOI:** 10.1371/journal.pone.0132742

**Published:** 2015-07-29

**Authors:** Katherine S. Lontok, Hubert Zhang, Michael J. Dougherty

**Affiliations:** 1 American Society of Human Genetics, Bethesda, Maryland, United States of America; 2 Department of Pediatrics, University of Colorado School of Medicine, Aurora, Colorado, United States of America; Institut Jacques Monod, FRANCE

## Abstract

Science standards have a long history in the United States and currently form the backbone of efforts to improve primary and secondary education in science, technology, engineering, and math (STEM). Although there has been much political controversy over the influence of standards on teacher autonomy and student performance, little light has been shed on how well standards cover science content. We assessed the coverage of genetics content in the Next Generation Science Standards (NGSS) using a consensus list of American Society of Human Genetics (ASHG) core concepts. We also compared the NGSS against state science standards. Our goals were to assess the potential of the new standards to support genetic literacy and to determine if they improve the coverage of genetics concepts relative to state standards. We found that expert reviewers cannot identify ASHG core concepts within the new standards with high reliability, suggesting that the scope of content addressed by the standards may be inconsistently interpreted. Given results that indicate that the disciplinary core ideas (DCIs) included in the NGSS documents produced by Achieve, Inc. clarify the content covered by the standards statements themselves, we recommend that the NGSS standards statements always be viewed alongside their supporting disciplinary core ideas. In addition, gaps exist in the coverage of essential genetics concepts, most worryingly concepts dealing with patterns of inheritance, both Mendelian and complex. Finally, state standards vary widely in their coverage of genetics concepts when compared with the NGSS. On average, however, the NGSS support genetic literacy better than extant state standards.

## Introduction

Standards in science education have a long history in the United States. In 1892, the National Education Association convened the Committee of Ten, consisting of leaders in secondary and higher education, to outline knowledge in nine broad subjects, including the sciences, that every graduating secondary school student should possess, regardless of that student’s future life course [1]. In the many standards-based reform movements since then, the philosophy behind educational standards has been that they promote equality of opportunity by providing every student with the same education regardless of circumstance [2].

In recent years, implementation of the science standards portion of the No Child Left Behind (NCLB) Act has led each U.S. state to enact its own set of science education standards. Although states have been required to assess students in science since the 2007–08 school year, inclusion of those results to demonstrate achievement of adequate yearly progress (AYP, a metric tied to school funding) remains optional [3]. Published analyses of content in state science standards have revealed a wide range of quality and content coverage [4–7], despite the fact that NCLB was intended to push states to provide an education of equal rigor for all students.

In 2011, the American Society of Human Genetics (ASHG) performed its own analysis of state science standards for coverage of genetics content using a list of ASHG core concepts [8]. These ASHG core concepts were developed by the ASHG education staff and the ASHG Information and Education Committee, whose members have expertise in both genetics content and science education, using previously published studies and documents defining content essential to basic genetic literacy–that is, the content that every graduating secondary school student should master to be well-prepared for encounters with genetics content in the public sphere (see [8]). Given ASHG’s focus as an organization and the fact that genetics in the public sphere is often centered on human health and disease, the ASHG core concepts are human-centric and do not encompass genetics concepts relevant only to other organisms.

As with other studies, our 2011 analysis using the ASHG core concepts found significant variability in state standards’ coverage of genetics concepts, and it found that few states covered the ASHG core concepts comprehensively [8]. Of the 19 ASHG core genetics concepts used to evaluate the state standards, only five concepts were, on average, adequately covered nationally; these concepts deal with the nature of genetic material, Mendelian inheritance patterns, and evolution. In addition, while two states covered 15 of the 19 ASHG core concepts adequately, some states adequately covered just one concept. The need for genetic literacy among the general public has only increased since our earlier analysis because of the rise of direct-to-consumer genetic testing, assisted-reproduction services, and the movement of genomics into mainstream healthcare, as reflected in the development of genetic testing guidelines by medical professional societies [9,10]. It is more important than ever for science education standards to include content critical to genetic literacy.

The Next Generation Science Standards (NGSS) were developed both as an update to the 1996 National Science Education Standards [11] and as a way to harmonize widely varying existing state science standards. The National Research Council developed the *Framework for K-12 Science Education* to outline scientific and engineering practices, crosscutting scientific concepts, and core ideas within specific disciplines (disciplinary core ideas or DCIs) that all students should master by the end of their secondary education [12]. Achieve, Inc., a non-profit organization, worked in collaboration with 26 lead states to develop the standards statements themselves in an iterative process, integrating all three dimensions of the *Framework* into performance expectations for students [13]. A representative of ASHG served as a “critical stakeholder” during the development of the standards by providing comments on early drafts that were not available for public comment. For more information on the NGSS development process, see [14]. In its final form, each performance expectation is supported in the NGSS document with references to the practice(s), crosscutting concept(s), and disciplinary core idea(s) integrated into that expectation. To date, twelve lead states have adopted the NGSS, including California, which has the largest public school student population in the U.S. [15–20]. In addition, Nevada and the District of Columbia, both uninvolved in the development of the NGSS, have also adopted them.

As states actively consider adopting the Next Generation Science Standards, now is the time to examine these new, potentially wide-reaching standards for discipline-specific content. Achieve, Inc., recently released the State Science Education Standards Comparison Tool, intended to assist state education officials in comparing their state standards with the NGSS [21]. This tool, however, asks broad questions about standard design and intent and does not provide questions intended to compare the robustness of disciplinary content. Thus, although this tool is important in helping potential users understand the structure of the new standards, it is not intended for content analysis.

The only analysis of the NGSS performed to date that includes evaluation of disciplinary content was done by The Thomas Fordham Institute [22]. The Institute awarded them an overall ‘C’ grade and ranked them as superior to the standards of 16 states, but inferior to the state standards of 12 states and the District of Columbia. Major issues cited by the Institute include missing content or content that is not explicit, the use of restrictive assessment boundaries, and the absence of math content necessary to support science content. The Institute’s analysis was not peer-reviewed, however, and given the Institute’s strong ideological views on the importance of learning scientific information over learning how to practice science, its analysis may be perceived as lacking objectivity [23]. Until now, there has been no objective analysis of the content covered by the new standards.

Using a process very similar to that used in our 2011 analysis of state science standards [8], we have evaluated the–Next Generation Science Standards for coverage of 19 ASHG core concepts in genetics. We found a disconcerting inability of expert reviewers to discern reliably how well these ASHG core concepts are represented within the NGSS. In addition, our analysis reveals that the NGSS have significant gaps in coverage of concepts related to modes of inheritance. With a few notable exceptions, however, we demonstrate that the NGSS cover genetics ASHG core concepts better than state standards did, on average, and that several states would likely improve their students’ genetic literacy were they to adopt the NGSS.

## Methods

### Collation of Next Generation Science Standards Pertaining to Genetics

To present reviewers with a manageable, yet inclusive, subset of the Next Generation Science Standards relevant to genetics, seven genetics experts (three ASHG staff and four members of the ASHG Information and Education Committee, all with terminal degrees in their field) reviewed all NGSS across all grade levels and identified those that were, in their view, “genetics-related” (see S1 File for the instructions we provided for this task). Fifteen standards were identified in common by all seven experts, and an additional three standards were identified in common by six of the seven experts. To be as comprehensive as possible, we chose to include in our final set any standard that was identified by at least two experts, which raised the total number of “genetics-related” standards to 28. Of those 28, seven were elementary school level, 11 were middle school level, and 10 were high school level. We excluded 14 'outlier' standards that were identified by only a single expert each (see S2 File for a listing of excluded and included NGSS standards). One expert in particular held an expansive view of “genetics-related” NGSS standards and was responsible for the identification of 11 of the 14 outlier standards.

We duplicated the verbatim text of the 28 identified standards (including any Clarification Statements and Assessment Boundaries [24]). We then assembled two PDF versions of the identified standards, either the standards statements alone (referred to throughout as “NGSS only,” S2 File) or in combination with referenced disciplinary core ideas from the NGSS document (not the NRC’s *Framework;* referred to throughout as “NGSS+DCI,” S2 File), which expand, to the largest extent possible, the amount of genetics content that might be considered by those implementing the new standards from the NGSS document alone.

### Recruitment of Volunteer Reviewers

We recruited volunteer reviewers by sending email invitations to ASHG members who self-identified as being interested in education, by targeting ASHG’s Genetics Education Outreach Network, and by reaching out to the pool of reviewers who participated in ASHG’s previous state standards analysis [8]. All respondents completed a brief demographic survey that included name, gender, address, institution, highest degree held, current position, comfort/experience level with science education standards in general, and previous participation in our analysis of state standards. Volunteers living outside of the U.S. were excluded from further consideration, for a total of 130 remaining volunteer reviewers. All remaining reviewers held at least a bachelor’s degree in the life sciences, with the large majority holding master’s (15%) or doctoral (81%) degrees.

### Pilot Study

We endeavored to develop a simple and intuitive evaluation process that required no training. To test our analysis parameters, we conducted a pilot study to evaluate inter-rater reliability among reviewers. From the full pool of 130 volunteer reviewers, we constructed two pilot groups of 20 reviewers each to proportionally match the full volunteer pool and each other across the following categories: 1) gender, 2) number of reviewers with a doctoral degree, 3) comfort/experience level with science education standards, and 4) previous experience as a state standards reviewer.

Using a custom online application, one group of pilot reviewers compared the ASHG core concepts (see [8] for a description of the development of these ASHG core concepts) to the standards statements alone (“NGSS only”), while the other group compared the ASHG core concepts to the standards statements in the context of references disciplinary core ideas (“NGSS+DCI”). Reviewers were instructed to read through the “NGSS only” or “NGSS+DCI” PDF at least once before performing any analysis (S3 File). Reviewers then were asked to read the first ASHG core concept and to revisit the “NGSS only” or “NGSS+DCI” document to analyze how well the *totality* of standards, or standards plus disciplinary core concepts, covered that concept. Reviewers then used a simple three-point ordinal scale to indicate coverage of the ASHG core concept: 0 = Not present (i.e., the ASHG core concept is not found in the set of NGSS standards, or NGSS standards and DCIs.), 1 = Present, inadequate (i.e., the ASHG core concept is found in the set of NGSS standards, or NGSS standards and DCIs, but there is a lack of completeness, specificity, clarity, accuracy, etc.), 2 = Present, adequate (i.e., the ASHG core concept is found in the set of NGSS standards, or NGSS standards and DCIs, in language that conveys the core concept’s essential elements.) (S3 File). This scale is identical to the scoring scale used in our state standards analysis [8]. Reviewers repeated this process for all remaining ASHG core concepts. In addition to scoring coverage of each ASHG core concept, reviewers had the option to provide written comments, although comments were not required. The analysis program provided the flexibility for reviewers to change their scores for any concept during any point in the analysis. Finally, reviewers were explicitly asked to review their scores for all 19 ASHG core concepts before submitting their analyses (S3 File).

Fifteen of the 20 reviewers in each pilot group completed their analyses within the 10-day pilot study window. We calculated an average score across all 19 ASHG core concepts for each reviewer. After confirming that the scores for both reviewer groups fit normal distributions as determined by measures of central tendency (e.g., mean, mode, skew), plots of frequency, and empirical distribution functions, we excluded any reviewer whose average score fell outside one standard deviation from the average score of his/her analysis group as outlier reviewers. We assessed inter-rater reliability by calculating Krippendorff’s alpha [25,26] across included reviewers for all 19 ASHG core concepts using ReCal OIR for ordinal data (Table 1) [27].

**Table 1:** Inter-rater reliability for pilot and full analysis reviewer groups.

Pilot analysis, reviewers of:	N*	Reliability*	N^	Reliability^
NGSS only	10	0.31	-	-
NGSS+DCI	12	0.52	-	-
**Full analysis, reviewers of:**				
NGSS only, odd concepts	9	0.56	11	0.56
NGSS only, even concepts	13	0.56	14	0.58
NGSS+DCI, odd concepts	8	0.60	9	0.73
NGSS+DCI, even concepts	11	0.57	10	0.62

All inter-rater reliabilities were measured using Krippendorff's alpha for ordinal data. *Includes only reviewers whose average scores (across all concepts) were within one standard deviation of the all-reviewer mean within each reviewer group.

^ Excludes concepts for which >20% of all reviewers deviated from the two most popular scores (i.e., high-disagreement concepts), and includes only reviewers whose average scores (across all included concepts) were within one standard deviation of the resulting all-reviewer mean within each reviewer group.

### Full Analysis

**Fig 1:**
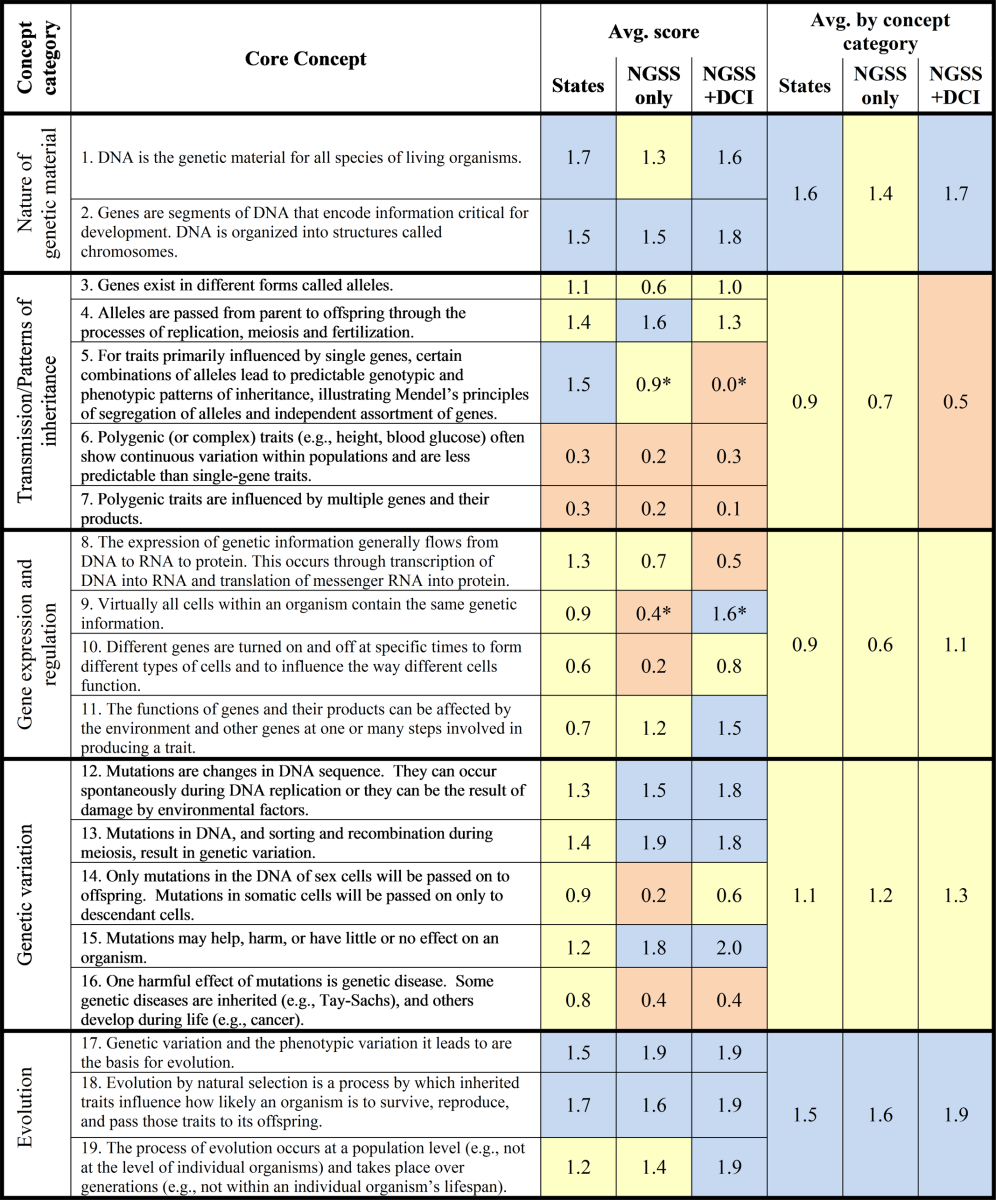
Average score for each ASHG genetics core concept and across concept categories within the “NGSS only” and the “NGSS+DCI,” compared to previous scores across state standards [8]. Numerical scores: 0–0.5 = Not present (orange); 0.6–1.4 = Present, inadequate (yellow); 1.5–2.0 = Present, adequate (blue). These rough bins correspond to score bins used in [8]. * indicates significant difference with a p-value <0.01 using Mann-Whitney rank score test.

Because of weak inter-rater reliability in the pilot analysis, we made two changes for the full analysis. First, to combat “reviewer fatigue,” reviewers were split into four groups rather than two: “NGSS only, even ASHG core concepts”; “NGSS only, odd ASHG core concepts”; “NGSS+DCI, even ASHG core concepts”; and “NGSS+DCI, odd ASHG core concepts” (“even” and “odd” refer to the numbering of the concepts in Fig 1). Concepts were divided by number rather than topic category (e.g., nature of genetic material, genetic variation) so that each group analyzed fewer concepts overall, but still considered a similar breadth of content. Each of the four groups contained 23 reviewers from the full reviewer pool, and groups were matched for select demographic characteristics as described in the pilot analysis. No reviewers from the pilot analysis participated in the full analysis.

Second, we introduced a training step in which reviewers scored three ASHG core concepts against the set of Alaska state standards evaluated in our previous state standards analysis [8]. Each concept chosen had been unanimously scored a 0, 1, or 2 in the previous analysis. To provide reviewers with explanations for “correct” (i.e., consensus) scores during training, one of us (KL, who did not participate in the analysis of state standards) underwent training in a blinded fashion, scored each concept, and developed a written rationale for each score, citing individual Alaska standards where appropriate (no explanatory comments were given by the previous state standards’ reviewers). In the final implementation of the training module, each reviewer entered his/her score for each concept and then received the consensus score given in the state standards analysis and our explanation for that score (S3 File).

We introduced no other changes between the pilot and full analysis. A total of 18 “NGSS only, even,” 15 “NGSS only, odd,” 16 “NGSS+DCI, even,” and 11 “NGSS+DCI, odd” reviewers completed their analyses within the 10-day full analysis window.

### Data Analysis

We conducted a normality check, as described in the pilot analysis, for each group of reviewers, and each group fit a normal distribution. We therefore calculated an average score across all analyzed concepts for each reviewer and compared to the group mean. Reviewers with average scores outside of one standard deviation from the group mean were excluded as outlier reviewers and Krippendorff’s alpha for ordinal data was calculated using ReCal OIR (Table 1).

To investigate the possibility that certain ASHG core concepts engendered more disagreement among reviewers than others, we tallied the number of reviewers that assigned a 0, 1, or 2 to each concept for each group of reviewers *before exclusion of outlier reviewers* (S1 Table). Concepts for which at least 20% of reviewers were two points away from the majority of reviewers (i.e., scored a concept 0 when most reviewers scored it 2) were considered high-disagreement concepts. We removed high-disagreement concepts from the raw data and reanalyzed, again excluding outlier reviewers from each group (outlier status calculated after concept exclusion; Table 1). The goal of this analysis was to determine the extent to which high-disagreement concepts did or did not affect overall reliability.

**Fig 2:**
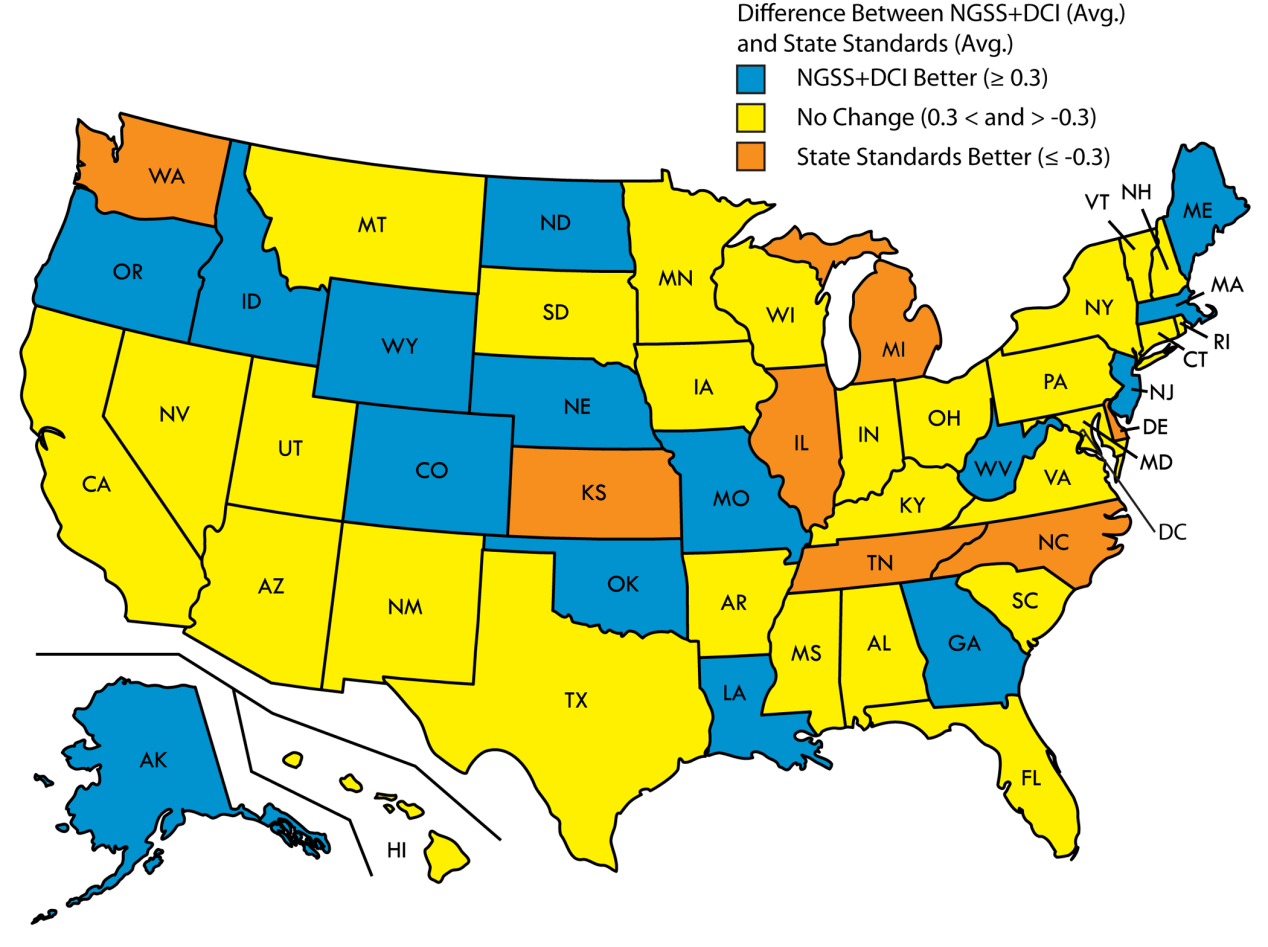
Map of the United States summarizing the difference in average coverage of ASHG core concepts between “NGSS+DCI” and each state’s standards (as determined in [8]). States shown in orange have a higher average state standards score for ASHG core concepts than “NGSS+DCI” (≤-0.3 difference). States shown in yellow have a comparable average state standards score for ASHG core concepts as “NGSS+DCI” (>-0.3 difference, but <0.3 difference). States shown in blue have a lower average state standards score for ASHG core concepts than “NGSS+DCI” (≥0.3 difference). Reprinted and modified from Wikimedia Commons under a CC BY license, with permission from Wikimedia Commons, original copyright 2007.

After calculating reliability, we conducted a complete analysis of the extent to which each concept was represented in the NGSS, looking at all concepts (including high-disagreement concepts) and using reviewers whose average scores were within one standard deviation of their group’s mean. Average scores were calculated for each ASHG core concept for “NGSS only” or “NGSS+DCI,” as well as across concept categories (Fig 1, binned according to categories from [8]). To compare the relationship between “NGSS only” average scores and “NGSS+DCI” average scores by concept, we calculated the correlation by Spearman’s rank sum. We used the Mann-Whitney rank sum test to determine if the average “NGSS only” versus “NGSS+DCI” scores for each concept were significantly different. We also compared average “NGSS only” or “NGSS+DCI” scores for each ASHG core concept to the average score for each concept across each state analyzed in the previous state standards analysis, as well as the average for each concept across all states (S2 and S3 Tables and Fig 1). We could not perform statistical comparisons between the NGSS and state standards data sets because of differences in study parameters. To avoid undue stringency, we set a minimum threshold of at least 0.3 in average score difference as our standard for meaningful differences in content coverage between the NGSS and state standards. Finally, the average “NGSS only” and “NGSS+DCI” scores across all concepts were similarly compared to each state’s average score across all concepts to gauge overall coverage of essential genetics content (Fig 2).

## Results

Inter-rater reliability is an important measure of the consistency with which different reviewers approach the same analysis. Ideal inter-rater reliabilities are 0.7 and higher [28]. In our pilot analysis, both reviewer groups, one scoring only the standards statements (“NGSS only”) and one scoring the standards statements in the context of referenced disciplinary core ideas (“NGSS+DCI”) against the ASHG core concepts, had inter-rater reliabilities lower than 0.7 (Table 1). Notably, those who saw disciplinary core ideas in addition to the standards statements had considerably better inter-rater reliability than those who did not.

Low inter-rater reliability prompted us to reduce the number of ASHG core concepts scored by each reviewer during the full analysis and to introduce a training module. Because of the similarity of this analysis to our previous state standards analysis, we chose to use examples from the state standards analysis as the basis for training. We chose to use the Alaska state standards in our training because there were ASHG core concepts that were unanimously scored 0, 1, or 2 against this particular set of standards in our previous analysis. Implementation of those two changes in the full analysis led to a marked increase in inter-rater reliability for reviewer groups that only saw the standards statements, but only a small increase for reviewer groups that saw the standards in the context of referenced disciplinary core ideas (Table 1).

To test the hypothesis that certain ASHG core concepts generated more disagreement among reviewers than others, we reanalyzed the raw data, tallying the number of 0, 1, and 2 scores given for each concept (S1 Table). In several cases, more than 20% of reviewers differed by two scoring steps from the majority of reviewers (i.e., a 0 and a 2 rather than 0 and 1, or 1 and 2). When these “high-disagreement” concepts were excluded from analysis, however, only one group surpassed 0.7 inter-rater reliability (Table 1). Because high-disagreement concepts did not appear to explain poor inter-rater reliability for three of the four analysis groups, we reverted to our previous analysis of the data, which included all ASHG core concepts.

By assigning reviewers to “NGSS only” and “NGSS+DCI” groups, we sought to determine if the presence of disciplinary core ideas influenced the apparent coverage of our ASHG core concepts. We averaged scores for each concept across reviewers for each group. By Spearman’s rank correlation, average scores by concept for “NGSS only” were strongly correlated with average scores by concept for “NGSS+DCI” (ρ = 0.78, p<0.001). Average “NGSS only” and “NGSS+DCI” scores were also compared using the Mann-Whitney rank sum test [29] (Fig 1). Although “NGSS+DCI” scores tended to be slightly higher, in most cases, “NGSS only” and “NGSS+DCI” scores for each ASHG core concept were not significantly different. However, in two cases, “NGSS only” and “NGSS+DCI” scores differed significantly (p<0.01) for the same ASHG core concept. Reviewers who only saw the standards statements scored ASHG core concept 5 significantly higher than those who saw the statements with their supporting disciplinary core ideas (“For traits primarily influenced by single genes, certain combinations of alleles lead to predictable genotypic and phenotypic patterns of inheritance, illustrating Mendel’s principles of segregation of alleles and independent assortment of genes”; Fig 1). In the second case, The standards statements in the context of disciplinary core ideas were scored significantly higher for ASHG core concept 9 than the standards statements alone (“Virtually all cells within an organism contain the same genetic information”).

To determine if the NGSS address individual concepts better or worse than they were addressed on average in state standards, we compared average “NGSS+DCI” scores for each concept to the average score across all states from our previous state standards analysis (Fig 1; [8]). We chose to use the “NGSS+DCI” scores for comparison rather than the scores for the standards statements alone because more ASHG core concepts were scored as adequately covered by reviewers who viewed the NGSS in context with the disciplinary core ideas. Because of differences between the study parameters, including the time elapsed between the two studies, the addition of a training step for the current analysis, and the use of reviewer pools that, while partially overlapping, may not have been demographically equivalent, we could not compare “NGSS+DCI” and state scores statistically. However, we set a score difference of 0.3 as the rough threshold at which we might expect to see meaningful differences in coverage of genetics concepts. Using that threshold, eight ASHG core concepts (2, 9, 11, 12, 13, 15, 17, and 19) are better covered by the new standards (when viewed with their supporting disciplinary core ideas) than they were by state standards, averaged nationally. ASHG core concepts 11 and 15, covering the concepts that traits can result from the combined effects of many genes and the environment and that mutations can have positive, negative, or no effect, showed the largest positive difference in scores between “NGSS+DCI” and the states’ standards (+0.8 difference).

In contrast, four ASHG core concepts (5, 8, 14, and 16) are not covered as well in the the new standards (when viewed with their supporting disciplinary core ideas) as they were in state standards. These concepts cover Mendelian genetics, the central dogma, the heritability of mutations found in germline versus somatic cells, and genetic disease as a possible effect of mutations. ASHG core concept 5, covering Mendelian inheritance, had the largest negative difference in score (-1.5 difference), and was in fact the only ASHG core concept to average a score of 0.0 for any of the comparisons made in this study (Fig 1). Only one reviewer out of 11 marked it as present, though inadequate (i.e. 1). The remaining seven ASHG core concepts (1, 3, 4, 6, 7, 10, and 18) are covered about as well in the “NGSS+DCI” as in state standards, averaged nationally.

Using the same 0.3 threshold, we also analyzed whether “NGSS+DCI” covered the ASHG core concepts better or worse than each individual state’s standards (S3 Table and Fig 2). The new standards (when viewed with their supporting disciplinary core ideas)cover the ASHG core concepts in aggregate better than the state standards in place in 2011 in 15 states, and about as well as state standards in 28 states. State standards for Delaware, Michigan, North Carolina, Tennessee, Illinois, Kansas, and Washington, however, covered ASHG core concepts, on average, better than “NGSS+DCI.” Perhaps not surprisingly, these seven states scored at the top of our prior analysis [8]. Looked at another way, 33 states adequately cover fewer ASHG core concepts than the NGSS (<10), while only 12 adequately cover more (S3 Table).

## Discussion

Our analysis is the first peer-reviewed study that examines the Next Generation Science Standards (NGSS) for inclusion of specific disciplinary content. We determined how well the new standards address concepts previously identified by the American Society of Human Genetics (ASHG) as constituting basic genetic literacy–that is, concepts all graduating secondary school students should master to be well-prepared for encounters with genetics in the public sphere [8]. We analyzed the NGSS standards when presented alone, or when presented in the context of their supporting disciplinary core ideas (DCIs), to understand the range of interpretation possible by stakeholders who will implement them. We also evaluated how well the NGSS address the ASHG core concepts in comparison to state standards in an attempt to determine if the NGSS represent a step forward in supporting genetic literacy.

Although we used an uncomplicated scoring system, a training module, and a study design that reduced reviewer workload, the reviewers in our final analysis did not achieve high levels of inter-rater reliability. We cannot rule out the possibility that insufficient training, imprecise interpretation of the scoring system, or a demographically heterogeneous reviewer pool contributed to less than ideal inter-rater reliability within each group. For instance, it is possible that more examples of each score in the training module would have led to more consistent scoring. It is important to note that elementary school (ES) standards were included in the genetics-related subset of NGSS standards that we provided to reviewers. Lower grade-level standards, in general, include less technical language, and it is possible that this broadened our judges’ interpretation of whether genetics-related concepts were addressed. Had we chosen to exclude the ES standards, inter-rater reliability might have improved, but the core concept scores may have converged on lower averages because of the loss of information contained in the ES standards.

Another potentially confounding factor in our analysis is the novel structure of the NGSS. Rather than being written as disciplinary facts that students must “know” or “understand” like many previous sets of science standards, the performance expectation statements used in the new standards assert what students should be able to do given mastery of the supporting scientific and engineering practices, crosscutting concepts, and disciplinary core ideas [24]. Because the NGSS are written as performance expectations rather than facts, it is possible that identification of the content addressed in the standards is inherently problematic and explains our modest inter-rater reliability. However, there are several reasons to believe that this is not the case. First, half of our reviewers saw the new standards in the context of supporting Disciplinary Core Ideas, which are not written as performance expectations and use “fact” language similar to traditional standards. Second, if the NGSS performance expectations are nebulous by nature, one would expect our reviewers to disagree with each other equally for all of the concepts we asked them to identify within the NGSS. Instead, some ASHG core concepts were scored much more consistently across reviewers than others. For example, the three ASHG core concepts in the “Evolution” concept category (core concepts 17, 18, and 19) were scored as “2, present, adequate” by all “NGSS+DCI” reviewers except one. Both “NGSS+DCI” reviewer groups were very clearly able to identify evolution concepts addressed by the NGSS, which demonstrates that performance expectations can, in fact, clearly communicate the underlying content that they are meant to address.

In total, six ASHG core concepts received unanimous or nearly unanimous scores (defined as a maximum of one dissenting score) from “NGSS+DCI” reviewers (S4 Table). Interestingly, these six ASHG core concepts were the two lowest-scoring concepts (core concepts 5 and 7) and four highest-scoring concepts (core concepts 15, 17, 18, and 19; Fig 1). Five of the six concepts with unanimous/nearly unanimous scores were reviewed by the “NGSS+DCI, odd core concepts” group. This distribution may explain why the “NGSS+DCI, odd core concepts” reviewer group achieved inter-rater reliability >0.7 when “high disagreement” concepts were removed, whereas the “NGSS+DCI, even core concepts” reviewer group did not. The fact that ASHG core concepts with unanimous/nearly unanimous scores cluster at both the bottom and the top of our scoring scale indicates that our reviewers largely agreed on content that was either entirely absent or fully covered. Ambiguity in the middle of the scale may have resulted from varied interpretations of the “1, present, inadequate” score (defined as “The ASHG core concept is found in the set of NGSS standards and DCIs, but there is a lack of completeness, specificity, clarity, accuracy, etc.” in the reviewer instructions, S3 File). However, it is also possible that reviewers varied in their interpretation of the standards themselves. For example, in reference to ASHG core concept 8 (average score of 0.5, not present from reviewers who saw the standards statements with the disciplinary core ideas), two reviewers who marked it “0, not present” commented on the absence of the term “RNA.” However, another reviewer, who also saw the standards statements with the disciplinary core ideas and scored ASHG core concept 8 as “1, present, inadequate” wrote, “…one may conclude that certain topics are implied by the proposed standard (e.g. the standards specify that DNA in genes codes for proteins, but the standards do not mention RNA as the intermediate—this might imply that the central dogma of DNA—RNA—to protein would be taught).” In this case, reviewers disagree in their interpretation of what the NGSS standards encompass rather than what our scores mean. The lack of explicit mention of RNA could lead to wide variation in how the central dogma is taught, from a mere mention that DNA encodes proteins, to a thorough exploration of transcription and translation, depending on whether the interpreter adheres to the letter of the standards or reads additional content into them. (We note that the National Research Council writes, in the introduction to LS3.A: Inheritance of Traits in the *Framework*, “DNA controls the expression of proteins by being transcribed into a “messenger” RNA, which is translated in turn by the cellular machinery into a protein” and presumably considers this level of detail to be appropriate for all students [12].) Because the new standards must be interpreted by numerous parties (such as curriculum developers, district science supervisors, and teachers) to be implemented, the apparent room left for widely differing interpretations, even when the disciplinary core ideas are present for context, may represent a serious challenge to implementation.

Although the official NGSS documents produced by Achieve, Inc. include the supporting disciplinary core ideas (as well as supporting science and engineering practices and crosscutting concepts), stakeholders implementing the standards may not take advantage of the additional context. For example, as curriculum developers produce materials aligned with the NGSS, they may focus on only the target standards statements and ignore the supporting material. In our analysis, “NGSS only” and “NGSS+DCI” scores for each ASHG core concept were not statistically different in most cases, but “NGSS+DCI” did score slightly higher than “NGSS only” for the majority of ASHG core concepts. This pattern could indicate that the supporting disciplinary core ideas either contain or more explicitly cover content that is either missing or implicit in the standards statements themselves. Several other observations support the importance of using the NGSS with the DCIs: 1) “NGSS only” reviewers had much lower inter-rater reliability than “NGSS+DCI” reviewers in the pilot analysis, which lacked a training step and may approximate implementation in many settings; 2) in the full analysis, “NGSS+DCI” reviewers scored six ASHG core concepts unanimously/nearly unanimously, whereas “NGSS only” reviewers gave unanimous/nearly unanimous scores to only three ASHG core concepts; and 3) reviewer comments for the two concepts that did receive statistically different “NGSS only” versus “NGSS+DCI” scores suggest that the DCIs serve an important role in explicitly clarifying the content addressed by the standards. In the case of ASHG core concept 5, covering Mendelian genetics, the standards statements alone actually scored significantly higher than “NGSS+DCI,” against the overall trend. However, one “NGSS+DCI” reviewer commented, “there is indication of using Punnett's squares to predict outcomes, but [the] focus seems to be on generation of variation, not predictable patterns. No mention of Mendel’s laws.” In this case, “NGSS only” reviewers may have given credit for seemingly implicit coverage of ASHG core concept 5 in the standards alone (where Punnett squares are mentioned) that could not be supported when viewed with the more concrete language of the corresponding disciplinary core ideas. In the second case, ASHG core concept 9 was scored significantly higher by reviewers who saw the standards statements in context with disciplinary core ideas versus “NGSS only” reviewers. In his/her comments, one “NGSS+DCI” reviewer specifically quotes part of disciplinary core idea LS3.A (“All cells in an organism have the same genetic content”) as rationale for scoring concept 9 as adequately addressed. No such explicit language is included in the standards themselves, perhaps explaining why “NGSS only” reviewers scored this concept as not present. Based on these results, we strongly recommend that the standards statements be interpreted in the context of their supporting disciplinary core ideas whenever possible.

As with state standards, the NGSS have both strengths and weaknesses in addressing genetics content. Ten of 19 total ASHG core concepts were scored as adequately addressed when the standards statements were viewed with disciplinary core ideas (ASHG core concepts 1, 2, 9, 11, 12, 13, 15, 17, 18, and 19; Fig 1), a sizable increase over the five ASHG core concepts that were adequately addressed, on average, across all state standards. These ten concepts encompass the nature of genetic material and evolution concept categories (which were also adequately covered, on average, in state standards), as well as select ASHG core concepts in the gene expression and regulation and genetic variation categories, which were, on average, inadequately covered in state standards. Although state standards, on average, covered evolution concepts well, it is noteworthy that the genetic basis of evolution is particularly well-covered in the new standards. All three evolution concepts (ASHG core concepts 17, 18, and 19) averaged almost perfect scores from “NGSS+DCI” reviewers. Given the attacks on the teaching of evolution currently in progress in some states, strong representation in the NGSS is a welcome statement on the centrality of evolution to the life sciences [30,31].

On the other hand, “NGSS+DCI” reviewers scored five ASHG core concepts, covering Mendelian genetics, complex trait genetics, gene expression and regulation, and mutations as the basis for genetic disease (core concepts 5, 6, 7, 8, and 16) as not only lacking in coverage, but absent altogether (Fig 1). Only two of these ASHG core concepts were not present, on average, in state standards (ASHG core concepts 6 and 7). Strikingly, ASHG core concept 5 (Mendelian genetics) was, in the state standards, one of the five best-addressed ASHG core concepts. Genetics education experts, including an author of this study (MJD), have previously argued that Mendelian genetics is too often over-emphasized to the detriment of a broader, more accurate understanding of genetics [32–34]. Mendel’s principles of segregation of alleles and independent assortment of genes are critical, however, to understanding how the behavior of chromosomes relates to inheritance. The fact that the NGSS do not address these principles at all is troubling. In addition, the ASHG core concepts dealing with polygenic (or complex) traits (ASHG core concepts 6 and 7), which one might reasonably expect to be emphasized in the absence of Mendelian genetics, are also lacking. An understanding of complex traits is more important to genetic literacy than ever as scientists strive to unravel the mechanisms by which both environmental and genetic factors influence most traits and to apply that understanding to human health. In total, the NGSS completely lack coverage of concepts necessary for understanding patterns of inheritance (discrete and continuous), one of the fundamental aims of the field of genetics.

At the level of individual states, our results offer no clear indication of whether adoption of the NGSS would result in improved coverage of genetics content for the majority of states. In 15 states, however, some of which were found to cover as few as one ASHG core concept adequately in their state standards, coverage of genetics content would likely improve if the state were to adopt the new standards (S3 Table and Fig 2). Unfortunately, of the 12 states and the District of Columbia that have already adopted NGSS [35–37], four (Delaware, Kansas, Illinois, and Washington) had superior coverage of genetics content in their state standards, according to our analyses, while three, Oregon, New Jersey, and West Virginia, had inferior coverage of genetics content in their state standards. Of the two states that have adopted budgets that prohibit the use of state funds to adopt NGSS (effectively rejecting the standards), South Carolina’s coverage of genetics content is comparable to the NGSS, while Wyoming’s coverage of genetics content is inferior to the NGSS [38–40]. Standards adoption has been politically charged, and several of the states that have poor coverage of genetics content in their state standards may choose not to adopt the NGSS and thus may not benefit from its potential to improve the genetic literacy of their students.

Given the results of this analysis, we believe that the NGSS represent a slight improvement in genetics content coverage nationally and a significant improvement in genetics content coverage for select states. However, many concepts essential to genetic literacy are either inadequately covered or simply not present in the NGSS, including Mendelian and complex trait genetics. Overall, our results suggest that stakeholders will need to supplement the NGSS with content recommendations from genetics experts in order for students to achieve true genetic literacy.

## Supporting Information

S1 File Instructions to experts identifying “genetics-related” NGSS standards. (PDF)

S2 File “Genetics-related” NGSS standards. The first set of standards were voted “genetics-related” by only one expert and were not included in the final set used by reviewers. “NGSS only” lists the final set of standards used by reviewers who analyzed the standards statements alone for coverage of ASHG core concepts. “NGSS+DCI” lists the final set of standards used by reviewers who analyzed the standards statements in the context of supporting Disciplinary Core Ideas for coverage of ASHG core concepts.
(PDF)

S3 File Example of instructions to reviewers from the “NGSS+DCI” pilot group. Reviewers in the “NGSS only” group received identical instructions except that all mentions of Disciplinary Core Ideas or DCIs were removed. Reviewers in the full analysis received identical instructions except for the addition of the following instructions after step 1: “2) Using the general method outlined below, complete the training analysis to familiarize yourself with the 0–2 scoring scale. 3) After completing the training analysis, note how your scores and rationale compare to the consensus scores of reviewers from our 2011 state standards analysis.”
(PDF)

S4 File Use license from the original copyright holder of Fig 2. (PDF)

S1 Table Number of reviewers in the “NGSS only” and “NGSS+DCI” groups, including “outlier” reviewers who were later excluded, who assigned each score 0–2 for each concept. Highlighted cells indicate “high-disagreement” concepts for each group.
(PDF)

S2 Table Difference between "NGSS only" and state standards by concept number. Difference was calculated as the average for "NGSS only" minus the state standards average [8] for each concept. Cells shaded orange indicate instances where the average "NGSS only" score is better by 0.3 or more. Blue shaded cells indicate instances where the average for the state standards is better by 0.3 or more.
(PDF)

S3 Table Difference between "NGSS+DCI" and state standards by concept number. Difference was calculated as the average for "NGSS+DCI" minus the state standards average [8] for each concept. Cells shaded purple indicate instances where the average "NGSS+DCI" score is better by 0.3 or more. Green shaded cells indicate instances where the average for the state standards is better by 0.3 or more.
(PDF)

S4 Table Number of included reviewers in the “NGSS only” and “NGSS+DCI” groups (excluding “outlier” reviewers) who assigned each score 0–2 for each concept. Highlighted cells indicate “unanimous/nearly unanimous” core concepts.
(PDF)
