# Supplementary material for: Assessing the Genetics Content in the Next Generation Science Standards
Source: PLoS One. 2015 Jul 29;10(7):e0132742. doi: 10.1371/journal.pone.0132742 (PMC4519196; doi:10.1371/journal.pone.0132742)
Supplement: S1 File — (PDF) [file pone.0132742.s001.pdf]

S1 Text. Instructions to experts identifying "genetics-related" NGSS standards.

The Task: Author KL, author MJD, and [third ASHG staff member] have independently gone through the NGSS document to identify any and all standards that deal with genetics in all its grade-appropriate forms-- inheritance, heredity, variation, reproduction, etc. However, we would like to have a larger number of experts do the same sort of independent identification so that we can collate the subset of genetics-related standards into the single, shorter PDF that reviewers will use during the actual analysis. In other words, we don't want every reviewer to need to read the entire NGSS when doing their evaluation; however, we need to ensure that we've captured all the essential standards. Your assistance helps us with this initial validation.

This process will likely take you 20-30 minutes, and you'll gain some knowledge about the organization and content of the NGSS. The attached document presents the standards in a horizontal box at the top of each page with three vertical boxes of context below. **We only need you to flag the standards themselves**. All you need to do is quickly read through the NGSS document and highlight the standard number (for example, MS-LS-1 or HS-LS-4) of the standards you think we should include by using Abode Reader/Acrobat's highlight function. Although the standards are grouped under headings that make it seem as if all will be genetics-related, such as "Heredity: Inheritance and Variation of Traits," you may disagree. Conversely, you may find standards within other groups, for example "From Molecules to Organisms: Structures and Processes," that you would consider to be genetic in nature. Thus, we request that you identify specific standards not whole categories.
