# Supplementary material for: Assessing the Genetics Content in the Next Generation Science Standards
Source: PLoS One. 2015 Jul 29;10(7):e0132742. doi: 10.1371/journal.pone.0132742 (PMC4519196; doi:10.1371/journal.pone.0132742)
Supplement: S2 File — The first set of standards were voted “genetics-related” by only one expert and were not included in the final set used by reviewers. “NGSS only” lists the final set of standards used by reviewers who analyzed the standards statements alone for coverage of ASHG core concepts. “NGSS+DCI” lists the final set of standards used by reviewers who analyzed the standards statements in the context of supporting Disciplinary Core Ideas for coverage of ASHG core concepts. (PDF) [file pone.0132742.s002.pdf]

NGSS Standards that received only one "genetics-related" vote and were thus excluded from further analysis:

K-ESS2-2. Construct an argument supported by evidence for how plants and animals (including humans) can change the environment to meet their needs. [Clarification Statement: Examples of plants and animals changing their environment coud include a squirrel digs in the ground to hide its food and tree roots can break concrete.]

K-ESS3-1. Use a model to represent the relationship between the needs of different plants or animals (including humans) and the places they live. [Clarification Statement: Examples of relationships could include that deer eat buds and leaves, therefore, they usually live in forested areas; and, grasses need sunlight so they often grow in meadows. Plants, animals, and their surroundings make up a system.]

1-LS1-2. Read texts and use media to determine patterns in behavior of parents and offspring that help offspring survive. [Clarification Statement: Examples of patterns of behaviors could include the signals that offspring make (such as crying, cheeping, and other vocalizations) and the responses of the parents (such as feeding, comforting, and protecting the offspring).]

2-ESS1-1. Make observations from media to construct an evidence-based account that Earth events can occur quickly or slowly. [Clarification Statement: Examples of events and timescales could include volcanic explosions and earthquakes, which happen quickly and erosion of rocks, which occurs slowly.] [Assessment Boundary: Assessment does not include quantitative measurements of timescales.]

3-LS4-4. Make a claim about the merit of a solution to a problem caused when the environment changes and the types of plants and animals that live there may change.* [Clarification Statement: Examples of environmental changes could include changes in land characteristics, water distribution, temperature, food, and other organisms.] [Assessment Boundary: Assessment is limited to a single environmental change. Assessment does not include the greenhouse effect or climate change.]

4-LS1-1. Construct an argument that plants and animals have internal and external structures that function to support survival, growth, behavior, and reproduction. [Clarification Statement: Examples of structures could include thorns, stems, roots, colored petals, heart, stomach, lung, brain, and skin.] [Assessment Boundary: Assessment is limited to macroscopic structures within plant and animal systems.]

5-PS3-1. Use models to describe that energy in animals' food (used for body repair, growth, motion, and to maintain body warmth) was once energy from the sun. [Clarification Statement: Examples of models could include diagrams, and flow charts.]

MS-PS1-1. Develop models to describe the atomic composition of simple molecules and extended structures. [Clarification Statement: Emphasis is on developing models of molecules that vary in complexity. Examples of simple molecules could include ammonia and methanol. Examples of extended structures could include sodium chloride or diamonds. Examples of molecular-level models could include drawings, 3D ball and stick structures, or computer representations showing different molecules with different types of atoms.] [Assessment Boundary: Assessment does not include valence electrons and bonding energy, discussing the ionic nature of subunits of complex structures, or a complete depiction of all individual atoms in a complex molecule or extended structure.]

MS-LS1-3. Use argument supported by evidence for how the body is a system of interacting subsystems composed of groups of cells. [Clarification Statement: Emphasis is on the conceptual understanding that cells form tissues and tissues form organs specialized for particular body functions. Examples could include the interaction of subsystems within a system and the normal functioning of those systems.] [Assessment Boundary: Assessment does not include the mechanism of one body system independent of others. Assessment is limited to the circulatory, excretory, digestive, respiratory, muscular, and nervous systems.]

MS-LS1-6. Construct a scientific explanation based on evidence for the role of photosynthesis in the cycling of matter and flow of energy into and out of organisms. [Clarification Statement: Emphasis is on tracing movement of matter and flow of energy.] [Assessment Boundary: Assessment does not include the biochemical mechanisms of photosynthesis.]

MS-LS1-8. Gather and synthesize information that sensory receptors respond to stimuli by sending messages to the brain for immediate behavior or storage as memories. [Assessment Boundary: Assessment does not include mechanisms for the transmission of this information.]

MS-LS2-1. Analyze and interpret data to provide evidence for the effects of resource availability on organisms and populations of organisms in an ecosystem. [Clarification Statement: Emphasis is on cause and effect relationships between resources and growth of individual organisms and the numbers of organisms in ecosystems during periods of abundant and scarce resources.]

MS-LS4-1. Analyze and interpret data for patterns in the fossil record that document the existence, diversity, extinction, and change of life forms throughout the history of life on Earth under the assumption that natural laws operate today as in the past. [Clarification Statement: Emphasis is on finding patterns of changes in the level of complexity of anatomical structures in organisms and the chronological order of fossil appearance in the rock layers.] [Assessment Boundary: Assessment does not include the names of individual species or geological eras in the fossil record.]

HS-LS1-6. Construct and revise an explanation based on evidence for how carbon, hydrogen, and oxygen from sugar molecules may combine with other elements to form amino acids and/or other large carbon-based molecules. [Clarification Statement: Emphasis is on using evidence from models and simulations to support explanations.] [Assessment Boundary: Assessment does not include the details of the specific chemical reactions or identification of macromolecules.]

HS-LS4-6. Create or revise a simulation to test a solution to mitigate adverse impacts of human activity on biodiversity.* [Clarification Statement: Emphasis is on designing solutions for a proposed problem related to threatened or endangered species, or to genetic variation of organisms for multiple species.]

"NGSS Only"

1-LS3-1. Make observations to construct an evidence-based account that young plants and animals are like, but not exactly like, their parents. [Clarification Statement: Examples of patterns could include features plants or animals share. Examples of observations could include leaves from the same kind of plant are the same shape but can differ in size; and, a particular breed of dog looks like its parents but is not exactly the same.] [Assessment Boundary: Assessment does not include inheritance or animals that undergo metamorphosis or hybrids.]

2-LS4-1. Make observations of plants and animals to compare the diversity of life in different habitats. [Clarification Statement: Emphasis is on the diversity of living things in each of a variety of different habitats.] [Assessment Boundary: Assessment does not include specific animal and plant names in specific habitats.]

3-LS1-1. Develop models to describe that organisms have unique and diverse life cycles but all have in common birth, growth, reproduction, and death. [Clarification Statement: Changes organisms go through during their life form a pattern.] [Assessment Boundary: Assessment of plant life cycles is limited to those of flowering plants. Assessment does not include details of human reproduction.]

3-LS3-1. Analyze and interpret data to provide evidence that plants and animals have traits inherited from parents and that variation of these traits exists in a group of similar organisms. [Clarification Statement: Patterns are the similarities and differences in traits shared between offspring and their parents, or among siblings. Emphasis is on organisms other than humans.] [Assessment Boundary: Assessment does not include genetic mechanisms of inheritance and prediction of traits. Assessment is limited to non-human examples.]

3-LS3-2. Use evidence to support the explanation that traits can be influenced by the environment. [Clarification Statement: Examples of the environment affecting a trait could include normally tall plants grown with insufficient water are stunted; and, a pet dog that is given too much food and little exercise may become overweight.]

3-LS4-2. Use evidence to construct an explanation for how the variations in characteristics among individuals of the same species may provide advantages in surviving, finding mates, and reproducing. [Clarification Statement: Examples of cause and effect relationships could be plants that have larger thorns than other plants may be less likely to be eaten by predators; and, animals that have better camouflage coloration than other animals may be more likely to survive and therefore more likely to leave offspring.]

3-LS4-3. Construct an argument with evidence that in a particular habitat some organisms can survive well, some survive less well, and some cannot survive at all. [Clarification Statement: Examples of evidence could include needs and characteristics of the organisms and habitats involved. The organisms and their habitat make up a system in which the parts depend on each other.]

MS-LS1-1. Conduct an investigation to provide evidence that living things are made of cells; either one cell or many different numbers and types of cells. [Clarification Statement: Emphasis is on developing evidence that living things are made of cells, distinguishing between living and non-living cells, and understanding that living things may be made of one cell or many and varied cells.]

MS-LS1-2. Develop and use a model to describe the function of a cell as a whole and ways parts of cells contribute to the function. [Clarification Statement: Emphasis is on the cell functioning as a whole system and the primary role of identified parts of the cell, specifically the nucleus, chloroplasts, mitochondria, cell membrane, and cell wall.] [Assessment Boundary: Assessment of organelle structure/function relationships is limited to the cell wall and cell membrane. Assessment of the function of the other organelles is limited to their relationship to the whole cell. Assessment does not include the biochemical function of cells or cell parts.]

MS-LS1-4. Use argument based on empirical evidence and scientific reasoning to support an explanation for how characteristic animal behaviors and specialized plant structures affect the probability of successful reproduction of animals and plants respectively. [Clarification Statement: Examples of behaviors that affect the probability of animal reproduction could include nest building to protect young from cold, herding of animals to protect young from predators, and vocalization of animals and colorful plumage to attract mates for breeding. Examples of animal behaviors that affect the probability of plant reproduction could include transferring pollen or seeds, and creating conditions for seed germination and growth. Examples of plant structures could include bright flowers attracting butterflies that transfer pollen, flower nectar and odors that attract insects that transfer pollen, and hard shells on nuts that squirrels bury.]

MS-LS1-5. Construct a scientific explanation based on evidence for how environmental and genetic factors influence the growth of organisms. [Clarification Statement: Examples of local environmental conditions could include availability of food, light, space, and water. Examples of genetic factors could include large breed cattle and species of grass affecting growth of organisms. Examples of evidence could include drought decreasing plant growth, fertilizer increasing plant growth, different varieties of plant seeds growing at different rates in different conditions, and fish growing larger in large ponds than they do in small ponds.] [Assessment Boundary: Assessment does not include genetic mechanisms, gene regulation, or biochemical processes.]

MS-LS3-1. Develop and use a model to describe why structural changes to genes (mutations) located on chromosomes may affect proteins and may result in harmful, beneficial, or neutral effects to the structure and function of the organism. [Clarification Statement: Emphasis is on conceptual understanding that changes in genetic material may result in making different proteins.] [Assessment Boundary: Assessment does not include specific changes at the molecular level, mechanisms for protein synthesis, or specific types of mutations.]

MS-LS3-2. Develop and use a model to describe why asexual reproduction results in offspring with identical genetic information and sexual reproduction results in offspring with genetic variation. [Clarification Statement: Emphasis is on using models such as Punnett squares, diagrams, and simulations to describe the cause and effect relationship of gene transmission from parent(s) to offspring and resulting genetic variation.]

MS-LS4-2. Apply scientific ideas to construct an explanation for the anatomical similarities and differences among modern organisms and between modern and fossil organisms to infer evolutionary relationships. [Clarification Statement: Emphasis is on explanations of the evolutionary relationships among organisms in terms of similarity or differences of the gross appearance of anatomical structures.]

MS-LS4-3. Analyze displays of pictorial data to compare patterns of similarities in the embryological development across multiple species to identify relationships not evident in the fully formed anatomy. [Clarification Statement: Emphasis is on inferring general patterns of relatedness among embryos of different organisms by comparing the macroscopic appearance of diagrams or pictures.] [Assessment Boundary: Assessment of comparisons is limited to gross appearance of anatomical structures in embryological development.]

MS-LS4-4. Construct an explanation based on evidence that describes how genetic variations of traits in a population increase some individuals' probability of surviving and reproducing in a specific environment. [Clarification Statement: Emphasis is on using simple probability statements and proportional reasoning to construct explanations.]

MS-LS4-5. Gather and synthesize information about the technologies that have changed the way humans influence the inheritance of desired traits in organisms. [Clarification Statement: Emphasis is on synthesizing information from reliable sources about the influence of humans on genetic outcomes in artificial selection (such as genetic modification, animal husbandry, gene therapy); and, on the impacts these technologies have on society as well as the technologies leading to these scientific discoveries.]

MS-LS4-6. Use mathematical representations to support explanations of how natural selection may lead to increases and decreases of specific traits in populations over time. [Clarification Statement: Emphasis is on using mathematical models, probability statements, and proportional reasoning to support explanations of trends in changes to populations over time.] [Assessment Boundary: Assessment does not include Hardy Weinberg calculations.]

HS-LS1-1. Construct an explanation based on evidence for how the structure of DNA determines the structure of proteins which carry out the essential functions of life through systems of specialized cells. [Assessment Boundary: Assessment does not include identification of specific cell or tissue types, whole body systems, specific protein structures and functions, or the biochemistry of protein synthesis.]

HS-LS1-4. Use a model to illustrate the role of cellular division (mitosis) and differentiation in producing and maintaining complex organisms. [Assessment Boundary: Assessment does not include specific gene control mechanisms or rote memorization of the steps of mitosis.]

HS-LS3-1. Ask questions to clarify relationships about the role of DNA and chromosomes in coding the instructions for characteristic traits passed from parents to offspring. [Assessment Boundary: Assessment does not include the phases of meiosis or the biochemical mechanism of specific steps in the process.]

HS-LS3-2. Make and defend a claim based on evidence that inheritable genetic variations may result from: (1) new genetic combinations through meiosis, (2) viable errors occurring during replication, and/or (3) mutations caused by environmental factors. [Clarification Statement: Emphasis is on using data to support arguments for the way variation occurs.] [Assessment Boundary: Assessment does not include the phases of meiosis or the biochemical mechanism of specific steps in the process.]

HS-LS3-3. Apply concepts of statistics and probability to explain the variation and distribution of expressed traits in a population. [Clarification Statement: Emphasis is on the use of mathematics to describe the probability of traits as it relates to genetic and environmental factors in the expression of traits.] [Assessment Boundary: Assessment does not include Hardy-Weinberg calculations.]

HS-LS4-1. Communicate scientific information that common ancestry and biological evolution are supported by multiple lines of empirical evidence. [Clarification Statement: Emphasis is on a conceptual understanding of the role each line of evidence has relating to common ancestry and biological evolution. Examples of evidence could include similarities in DNA sequences, anatomical structures, and order of appearance of structures in embryological development.]

HS-LS4-2. Construct an explanation based on evidence that the process of evolution primarily results from four factors: (1) the potential for a species to increase in number, (2) the heritable genetic variation of individuals in a species due to mutation and sexual reproduction, (3) competition for limited resources, and (4) the proliferation of those organisms that are better able to survive and reproduce in the environment. [Clarification Statement: Emphasis is on using evidence to explain the influence each of the four factors has on number of organisms, behaviors, morphology, or physiology in terms of ability to compete for limited resources and subsequent survival of individuals and adaptation of species. Examples of evidence could include mathematical models such as simple distribution graphs and proportional reasoning.] [Assessment Boundary: Assessment does not include other mechanisms of evolution, such as genetic drift, gene flow through migration, and co-evolution.]

HS-LS4-3. Apply concepts of statistics and probability to support explanations that organisms with an advantageous heritable trait tend to increase in proportion to organisms lacking this trait. [Clarification Statement: Emphasis is on analyzing shifts in numerical distribution of traits and using these shifts as evidence to support explanations.] [Assessment Boundary: Assessment is limited to basic statistical and graphical analysis. Assessment does not include allele frequency calculations.]

HS-LS4-4. Construct an explanation based on evidence for how natural selection leads to adaptation of populations. [Clarification Statement: Emphasis is on using data to provide evidence for how specific biotic and abiotic differences in ecosystems (such as ranges of seasonal temperature, long-term climate change, acidity, light, geographic barriers, or evolution of other organisms) contribute to a change in gene frequency over time, leading to adaptation of populations.]

HS-LS4-5. Evaluate the evidence supporting claims that changes in environmental conditions may result in: (1) increases in the number of individuals of some species, (2) the emergence of new species over time, and (3) the extinction of other species. [Clarification Statement: Emphasis is on determining cause and effect relationships for how changes to the environment such as deforestation, fishing, application of fertilizers, drought, flood, and the rate of change of the environment affect distribution or disappearance of traits in species.]

"NGSS+DCI"

1-LS3-1. Make observations to construct an evidence-based account that young plants and animals are like, but not exactly like, their parents. [Clarification Statement: Examples of patterns could include features plants or animals share. Examples of observations could include leaves from the same kind of plant are the same shape but can differ in size; and, a particular breed of dog looks like its parents but is not exactly the same.] [Assessment Boundary: Assessment does not include inheritance or animals that undergo metamorphosis or hybrids.]

- Young animals are very much, but not exactly, like their parents. Plants also are very much, but not exactly, like their parents. (LS3.A: Inheritance of Traits)
- Individuals of the same kind of plant or animal are recognizable as similar but can also vary in many ways. (LS3.B: Variation of Traits)

2-LS4-1. Make observations of plants and animals to compare the diversity of life in different habitats. [Clarification Statement: Emphasis is on the diversity of living things in each of a variety of different habitats.] [Assessment Boundary: Assessment does not include specific animal and plant names in specific habitats.]

- There are many different kinds of living things in any area, and they exist in different places on land and in water. (LS4.D: Biodiversity and Humans)

3-LS1-1. Develop models to describe that organisms have unique and diverse life cycles but all have in common birth, growth, reproduction, and death. [Clarification Statement: Changes organisms go through during their life form a pattern.] [Assessment Boundary: Assessment of plant life cycles is limited to those of flowering plants. Assessment does not include details of human reproduction.]

- Reproduction is essential to the continued existence of every kind of organism. Plants and animals have unique and diverse life cycles. (LS1.B: Growth and Development of Organisms)

3-LS3-1. Analyze and interpret data to provide evidence that plants and animals have traits inherited from parents and that variation of these traits exists in a group of similar organisms. [Clarification Statement: Patterns are the similarities and differences in traits shared between offspring and their parents, or among siblings. Emphasis is on organisms other than humans.] [Assessment Boundary: Assessment does not include genetic mechanisms of inheritance and prediction of traits. Assessment is limited to non-human examples.]

- Many characteristics of organisms are inherited from their parents. (LS3.A: Inheritance of Traits)
- Different organisms vary in how they look and function because they have different inherited information. (LS3.B: Variation of Traits)

3-LS3-2. Use evidence to support the explanation that traits can be influenced by the environment. [Clarification Statement: Examples of the environment affecting a trait could include normally tall plants grown with insufficient water are stunted; and, a pet dog that is given too much food and little exercise may become overweight.]

- Other characteristics result from individuals' interactions with the environment, which can range from diet to learning. Many characteristics involve both inheritance and environment. (LS3.A: Inheritance of Traits)
- The environment also affects the traits that an organism develops. (LS3.B: Variation of Traits)

3-LS4-2. Use evidence to construct an explanation for how the variations in characteristics among individuals of the same species may provide advantages in surviving, finding mates, and reproducing. [Clarification Statement: Examples of cause and effect relationships could be plants that have larger thorns than other plants may be less likely to be eaten by predators; and, animals that have better camouflage coloration than other animals may be more likely to survive and therefore more likely to leave offspring.]

- Sometimes the differences in characteristics between individuals of the same species provide advantages in surviving, finding mates, and reproducing. (LS4.B: Natural Selection)

3-LS4-3. Construct an argument with evidence that in a particular habitat some organisms can survive well, some survive less well, and some cannot survive at all. [Clarification Statement: Examples of evidence could include needs and characteristics of the organisms and habitats involved. The organisms and their habitat make up a system in which the parts depend on each other.]

- For any particular environment, some kinds of organisms survive well, some survive less well, and some cannot survive at all. (LS4.C: Adaptation)

MS-LS1-1. Conduct an investigation to provide evidence that living things are made of cells; either one cell or many different numbers and types of cells. [Clarification Statement: Emphasis is on developing evidence that living things are made of cells, distinguishing between living and non-living cells, and understanding that living things may be made of one cell or many and varied cells.]

- All living things are made up of cells, which is the smallest unit that can be said to be alive. An organism may consist of one single cell (unicellular) or many different numbers and types of cells (multicellular). (LS1.A: Structure and Function)

MS-LS1-2. Develop and use a model to describe the function of a cell as a whole and ways parts of cells contribute to the function. [Clarification Statement: Emphasis is on the cell functioning as a whole system and the primary role of identified parts of the cell, specifically the nucleus, chloroplasts, mitochondria, cell membrane, and cell wall.] [Assessment Boundary: Assessment of organelle structure/function relationships is limited to the cell wall and cell membrane. Assessment of the function of the other organelles is limited to their relationship to the whole cell. Assessment does not include the biochemical function of cells or cell parts.]

- Within cells, special structures are responsible for particular functions, and the cell membrane forms the boundary that controls what enters and leaves the cell. (LS1.A: Structure and Function)

MS-LS1-4. Use argument based on empirical evidence and scientific reasoning to support an explanation for how characteristic animal behaviors and specialized plant structures affect the probability of successful reproduction of animals and plants respectively. [Clarification Statement: Examples of behaviors that affect the probability of animal reproduction could include nest building to protect young from cold, herding of animals to protect young from predators, and vocalization of animals and colorful plumage to attract mates for breeding. Examples of animal behaviors that affect the probability of plant reproduction could include transferring pollen or seeds, and creating conditions for seed germination and growth. Examples of plant structures could include bright flowers attracting butterflies that transfer pollen, flower nectar and odors that attract insects that transfer pollen, and hard shells on nuts that squirrels bury.]

- Animals engage in characteristic behaviors that increase the odds of reproduction. (LS1.B: Growth and Development of Organisms)
- Plants reproduce in a variety of ways, sometimes depending on animal behavior and specialized features for reproduction. (LS1.B: Growth and Development of Organisms)

MS-LS1-5. Construct a scientific explanation based on evidence for how environmental and genetic factors influence the growth of organisms. [Clarification Statement: Examples of local environmental conditions could include availability of food, light, space, and water. Examples of genetic factors could include large breed cattle and species of grass affecting growth of organisms. Examples of evidence could include drought decreasing plant growth, fertilizer increasing plant growth, different varieties of plant seeds growing at different rates in different conditions, and fish growing larger in large ponds than they do in small ponds.] [Assessment Boundary: Assessment does not include genetic mechanisms, gene regulation, or biochemical processes.]

- Genetic factors as well as local conditions affect the growth of the adult plant. (LS1.B: Growth and Development of Organisms)

MS-LS3-1. Develop and use a model to describe why structural changes to genes (mutations) located on chromosomes may affect proteins and may result in harmful, beneficial, or neutral effects to the structure and function of the organism. [Clarification Statement: Emphasis is on conceptual understanding that changes in genetic material may result in making different proteins.] [Assessment Boundary: Assessment does not include specific changes at the molecular level, mechanisms for protein synthesis, or specific types of mutations.]

- Genes are located in the chromosomes of cells, with each chromosome pair containing two variants of each of many distinct genes. Each distinct gene chiefly controls the production of specific proteins, which in turn affects the traits of the individual. Changes (mutations) to genes can result in changes to proteins, which can affect the structures and functions of the organism and thereby change traits. (LS3.A: Inheritance of Traits)
- In addition to variations that arise from sexual reproduction, genetic information can be altered because of mutations. Though rare, mutations may result in changes to the structure and function of proteins. Some changes are beneficial, others harmful, and some neutral to the organism. (LS3.B: Variation of Traits)

MS-LS3-2. Develop and use a model to describe why asexual reproduction results in offspring with identical genetic information and sexual reproduction results in offspring with genetic variation. [Clarification Statement: Emphasis is on using models such as Punnett squares, diagrams, and simulations to describe the cause and effect relationship of gene transmission from parent(s) to offspring and resulting genetic variation.]

- Organisms reproduce, either sexually or asexually, and transfer their genetic information to their offspring. (LS1.B: Growth and Development of Organisms)
- Variations of inherited traits between parent and offspring arise from genetic differences that result from the subset of chromosomes (and therefore genes) inherited. (LS3.A: Inheritance of Traits)
- In sexually reproducing organisms, each parent contributes half of the genes acquired (at random) by the offspring. Individuals have two of each chromosome and hence two alleles of each gene, one acquired from each parent. These versions may be identical or may differ from each other. (LS3.B: Variation of Traits)

MS-LS4-2. Apply scientific ideas to construct an explanation for the anatomical similarities and differences among modern organisms and between modern and fossil organisms to infer evolutionary relationships. [Clarification Statement: Emphasis is on explanations of the evolutionary relationships among organisms in terms of similarity or differences of the gross appearance of anatomical structures.]

- Anatomical similarities and differences between various organisms living today and between them and organisms in the fossil record, enable the reconstruction of evolutionary history and the inference of lines of evolutionary descent. (LS4.A: Evidence of Common Ancestry and Diversity)

MS-LS4-3. Analyze displays of pictorial data to compare patterns of similarities in the embryological development across multiple species to identify relationships not evident in the fully formed anatomy. [Clarification Statement: Emphasis is on inferring general patterns of relatedness among embryos of different organisms by comparing the macroscopic appearance of diagrams or pictures.] [Assessment Boundary: Assessment of comparisons is limited to gross appearance of anatomical structures in embryological development.]

- Comparison of the embryological development of different species also reveals similarities that show relationships not evident in the fully-formed anatomy. (LS4.A: Evidence of Common Ancestry and Diversity)

MS-LS4-4. Construct an explanation based on evidence that describes how genetic variations of traits in a population increase some individuals' probability of surviving and reproducing in a specific environment. [Clarification Statement: Emphasis is on using simple probability statements and proportional reasoning to construct explanations.]

- Natural selection leads to the predominance of certain traits in a population, and the suppression of others. (LS4.B: Natural Selection)

MS-LS4-5. Gather and synthesize information about the technologies that have changed the way humans influence the inheritance of desired traits in organisms. [Clarification Statement: Emphasis is on synthesizing information from reliable sources about the influence of humans on genetic outcomes in artificial selection (such as genetic modification, animal husbandry, gene therapy); and, on the impacts these technologies have on society as well as the technologies leading to these scientific discoveries.]

- In artificial selection, humans have the capacity to influence certain characteristics of organisms by selective breeding. One can choose desired parental traits determined by genes, which are then passed on to offspring. (LS4.B: Natural Selection)

MS-LS4-6. Use mathematical representations to support explanations of how natural selection may lead to increases and decreases of specific traits in populations over time. [Clarification Statement: Emphasis is on using mathematical models, probability statements, and proportional reasoning to support explanations of trends in changes to populations over time.] [Assessment Boundary: Assessment does not include Hardy Weinberg calculations.]

- Adaptation by natural selection acting over generations is one important process by which species change over time in response to changes in environmental conditions. Traits that support successful survival and reproduction in the new environment become more common; those that do not become less common. Thus, the distribution of traits in a population changes. (LS4.C: Adaptation)

HS-LS1-1. Construct an explanation based on evidence for how the structure of DNA determines the structure of proteins which carry out the essential functions of life through systems of specialized cells. [Assessment Boundary: Assessment does not include identification of specific cell or tissue types, whole body systems, specific protein structures and functions, or the biochemistry of protein synthesis.]

- Systems of specialized cells within organisms help them perform the essential functions of life. (LS1.A: Structure and Function)
- All cells contain genetic information in the form of DNA molecules. Genes are regions in the DNA that contain the instructions that code for the formation of proteins, which carry out most of the work of cells. (LS1.A: Structure and Function)

HS-LS1-4. Use a model to illustrate the role of cellular division (mitosis) and differentiation in producing and maintaining complex organisms. [Assessment Boundary: Assessment does not include specific gene control mechanisms or rote memorization of the steps of mitosis.]

- In multicellular organisms individual cells grow and then divide via a process called mitosis, thereby allowing the organism to grow. The organism begins as a single cell (fertilized egg) that divides successively to produce many cells, with each parent cell passing identical genetic material (two variants of each chromosome pair) to both daughter cells. Cellular division and differentiation produce and maintain a complex organism, composed of systems of tissues and organs that work together to meet the needs of the whole organism. (LS1.B: Growth and Development of Organisms)

HS-LS3-1. Ask questions to clarify relationships about the role of DNA and chromosomes in coding the instructions for characteristic traits passed from parents to offspring. [Assessment Boundary: Assessment does not include the phases of meiosis or the biochemical mechanism of specific steps in the process.]

- All cells contain genetic information in the form of DNA molecules. Genes are regions in the DNA that contain the instructions that code for the formation of proteins. (LS1.A: Structure and Function)
- Each chromosome consists of a single very long DNA molecule, and each gene on the chromosome is a particular segment of that DNA. The instructions for forming species' characteristics are carried in DNA. All cells in an organism have the same genetic content, but the genes used (expressed) by the cell may be regulated in different ways. Not all DNA codes for a protein; some segments of DNA are involved in regulatory or structural functions, and some have no as-yet known function. (LS3.A: Inheritance of Traits)

HS-LS3-2. Make and defend a claim based on evidence that inheritable genetic variations may result from: (1) new genetic combinations through meiosis, (2) viable errors occurring during replication, and/or (3) mutations caused by environmental factors. [Clarification Statement: Emphasis is on using data to support arguments for the way variation occurs.] [Assessment Boundary: Assessment does not include the phases of meiosis or the biochemical mechanism of specific steps in the process.]

- In sexual reproduction, chromosomes can sometimes swap sections during the process of meiosis (cell division), thereby creating new genetic combinations and thus more genetic variation. Although DNA replication is tightly regulated and remarkably accurate, errors do occur and result in mutations, which are also a source of genetic variation. Environmental factors can also cause mutations in genes, and viable mutations are inherited. (LS3.B: Variation of Traits)
- Environmental factors also affect expression of traits, and hence affect the probability of occurrences of traits in a population. Thus the variation and distribution of traits observed depends on both genetic and environmental factors. (LS3.B: Variation of Traits)

HS-LS3-3. Apply concepts of statistics and probability to explain the variation and distribution of expressed traits in a population. [Clarification Statement: Emphasis is on the use of mathematics to describe the probability of traits as it relates to genetic and environmental factors in the expression of traits.] [Assessment Boundary: Assessment does not include Hardy-Weinberg calculations.]

- Environmental factors also affect expression of traits, and hence affect the probability of occurrences of traits in a population. Thus the variation and distribution of traits observed depends on both genetic and environmental factors. (LS3.B: Variation of Traits)

HS-LS4-1. Communicate scientific information that common ancestry and biological evolution are supported by multiple lines of empirical evidence. [Clarification Statement: Emphasis is on a conceptual understanding of the role each line of evidence has relating to common ancestry and biological evolution. Examples of evidence could include similarities in DNA sequences, anatomical structures, and order of appearance of structures in embryological development.]

- Genetic information provides evidence of evolution. DNA sequences vary among species, but there are many overlaps; in fact, the ongoing branching that produces multiple lines of descent can be inferred by comparing the DNA sequences of different organisms. Such information is also derivable from the similarities and differences in amino acid sequences and from anatomical and embryological evidence. (LS4.A: Evidence of Common Ancestry and Diversity)

HS-LS4-2. Construct an explanation based on evidence that the process of evolution primarily results from four factors: (1) the potential for a species to increase in number, (2) the heritable genetic variation of individuals in a species due to mutation and sexual reproduction, (3) competition for limited resources, and (4) the proliferation of those organisms that are better able to survive and reproduce in the environment. [Clarification Statement: Emphasis is on using evidence to explain the influence each of the four factors has on number of organisms, behaviors, morphology, or physiology in terms of ability to compete for limited resources and subsequent survival of individuals and adaptation of species. Examples of evidence could include mathematical models such as simple distribution graphs and proportional reasoning.] [Assessment Boundary: Assessment does not include other mechanisms of evolution, such as genetic drift, gene flow through migration, and co-evolution.]

- Natural selection occurs only if there is both (1) variation in the genetic information between organisms in a population and (2) variation in the expression of that genetic information—that is, trait variation—that leads to differences in performance among individuals. (LS4.B: Natural Selection)
- Evolution is a consequence of the interaction of four factors: (1) the potential for a species to increase in number, (2) the genetic variation of individuals in a species due to mutation and sexual reproduction, (3) competition for an environment's limited supply of the resources that individuals need in order to survive and reproduce, and (4) the ensuing proliferation of those organisms that are better able to survive and reproduce in that environment. (LS4.C: Adaptation)

HS-LS4-3. Apply concepts of statistics and probability to support explanations that organisms with an advantageous heritable trait tend to increase in proportion to organisms lacking this trait. [Clarification Statement: Emphasis is on analyzing shifts in numerical distribution of traits and using these shifts as evidence to support explanations.] [Assessment Boundary: Assessment is limited to basic statistical and graphical analysis. Assessment does not include allele frequency calculations.]

- Natural selection occurs only if there is both (1) variation in the genetic information between organisms in a population and (2) variation in the expression of that genetic information—that is, trait variation—that leads to differences in performance among individuals. (LS4.B: Natural Selection)
- The traits that positively affect survival are more likely to be reproduced, and thus are more common in the population. (LS4.B: Natural Selection)
- Natural selection leads to adaptation, that is, to a population dominated by organisms that are anatomically, behaviorally, and physiologically well suited to survive and reproduce in a specific environment. That is, the differential survival and reproduction of organisms in a population that have an advantageous heritable trait leads to an increase in the proportion of individuals in future generations that have the trait and to a decrease in the proportion of individuals that do not. (LS4.C: Adaptation)
- Adaptation also means that the distribution of traits in a population can change when conditions change. (LS4.C: Adaptation)

HS-LS4-4. Construct an explanation based on evidence for how natural selection leads to adaptation of populations. [Clarification Statement: Emphasis is on using data to provide evidence for how specific biotic and abiotic differences in ecosystems (such as ranges of seasonal temperature, long-term climate change, acidity, light, geographic barriers, or evolution of other organisms) contribute to a change in gene frequency over time, leading to adaptation of populations.]

- Natural selection leads to adaptation, that is, to a population dominated by organisms that are anatomically, behaviorally, and physiologically well suited to survive and reproduce in a specific environment. That is, the differential survival and reproduction of organisms in a population that have an advantageous heritable trait leads to an increase in the proportion of individuals in future generations that have the trait and to a decrease in the proportion of individuals that do not. (LS4.C: Adaptation)

HS-LS4-5. Evaluate the evidence supporting claims that changes in environmental conditions may result in: (1) increases in the number of individuals of some species, (2) the emergence of new species over time, and (3) the extinction of other species. [Clarification Statement: Emphasis is on determining cause and effect relationships for how changes to the environment such as deforestation, fishing, application of fertilizers, drought, flood, and the rate of change of the environment affect distribution or disappearance of traits in species.]

- Changes in the physical environment, whether naturally occurring or human induced, have thus contributed to the expansion of some species, the emergence of new distinct species as populations diverge under different conditions, and the decline–and sometimes the extinction–of some species. (LS4.C: Adaptation)
- Species become extinct because they can no longer survive and reproduce in their altered environment. If members cannot adjust to change that is too fast or drastic, the opportunity for the species' evolution is lost. (LS4.C: Adaptation)
