# Supplementary material for: Assessing the Genetics Content in the Next Generation Science Standards
Source: PLoS One. 2015 Jul 29;10(7):e0132742. doi: 10.1371/journal.pone.0132742 (PMC4519196; doi:10.1371/journal.pone.0132742)
Supplement: S3 File — Reviewers in the “NGSS only” group received identical instructions except that all mentions of Disciplinary Core Ideas or DCIs were removed. Reviewers in the full analysis received identical instructions except for the addition of the following instructions after step 1: “2) Using the general method outlined below, complete the training analysis to familiarize yourself with the 0–2 scoring scale. 3) After completing the training analysis, note how your scores and rationale compare to the consensus scores of reviewers from our 2011 state standards analysis.” (PDF) [file pone.0132742.s003.pdf]

Dear Colleague,

Thank you for volunteering to analyze the Next Generation Science Standards (NGSS). **The deadline to complete your analysis is August 23.**

<u>Objective</u>: To determine whether the NGSS encompass the 19 ASHG core genetics concepts identified as essential to genetic literacy in Dougherty et al. 2011. Please see the appendices for supplemental information on the background, rationale, and validation process.

<u>Instructions – **please read carefully**</u>:

1) Use the login information below to access the analysis site: http://www.ashg.org/education/natstdanalysis.shtml.
   Last Name:
   First Initial:
   Password:

2) In the top pane, read the 28 NGSS standards and their associated Disciplinary Core Ideas (DCIs) <u>once completely through</u> to familiarize yourself with the content BEFORE proceeding with the analysis. Note that only a validated "genetics" subset of the full NGSS standards has been included.

3) Read the first ASHG core concept in the lower pane.

4) In the top pane, read the 28 NGSS standards and the DCIs again and determine whether the ASHG concept is present and, if so, whether it is adequately or inadequately represented. A one-to-one match is not required; a core concept may be covered adequately across several standards. Use the definitions below.

0 - Not present	The ASHG core concept is not found in the set of NGSS standards and DCIs.
1 – Present, inadequate	The ASHG core concept is found in the set of NGSS standards and DCIs, but there is a lack of completeness, specificity, clarity, accuracy, etc.
2 – Present, adequate	The ASHG core concept is found in the set of NGSS standards and DCIs in language that conveys the core concept's essential elements.

Please use your best judgment to match concepts with standards and DCIs and do not assume too much. An incomplete standard that evokes for you (as an expert in genetics) a natural elaboration encompassing the ASHG core concept may not evoke that same connection for a non-expert. We need to evaluate what each standard actually says. At the same time, if the

intent of a standard clearly matches the intent of an ASHG core concept and differs only in wording, you should credit that standard appropriately.

5) Use the drop-down menu to the left of the first core concept to register your score. Record any comments by scrolling down to the comment box at the bottom of the lower pane.

6) Proceed to the second ASHG concept and repeat the process until you have evaluated the standards and DCIs with respect to all 19 ASHG core concepts. If you would like to continue your analysis later, you may save your scores by clicking “Save Your Scores” at the bottom of the page.

7) If at any point you would like to modify the score for a core concept, you may use the corresponding drop-down menu to do so.

8) Finally, review the scores you have assigned each ASHG core concept. If you would like to modify a score, use the corresponding drop-down menu to do so. When you are satisfied with your analysis, click “Submit You Final Scores”.

Please let us know if you encounter any technical difficulties. However, we cannot provide input on the analysis itself. Thank you again for your help.

Sincerely,

Michael J. Dougherty, Ph.D.
Director of Education, ASHG

Katherine Lontok, Ph.D.
Educational Programs Manager

Appendix A - Background and Rationale

The NGSS are voluntary standards that were developed from the document *A Framework for K-12 Science Education: Practices, Crosscutting Concepts, and Core Ideas* (2012), which was produced by the National Research Council (NRC). Achieve, Inc.--on a contract from the NRC and in conjunction with the National Association of Science Teachers, the Association for the Advancement of Science, and over 20 states--used the *Framework* as the guiding document to produce the NGSS through an iterative process lasting over a year. Unlike earlier standards, which were adapted in myriad ways by states, the NGSS are being treated as "take-it-or-leave-it" guidance for states, building on the process used for adoption of the Common Core standards in math and language arts, which were promoted to (and adopted or not) by governors. The intent is to prevent dilution, alteration, and omission of concepts viewed as essential by the science education community. More details can be found here: http://www.nextgenscience.org/.

Three years ago, ASHG provided a service to the science education community when it analyzed the genetics content of the current generation of standards, which were developed by individual states. That analysis was published in 2011 (Dougherty, M.J., Pleasants, C., Solow, L., Wong, A., and Zhang, H. A comprehensive analysis of high school genetics standards: Are states keeping pace with modern genetics? *CBE-Life Sciences Education* 10, 318-327.) Now you will help us in a complementary effort by evaluating the NGSS. The results of this analysis will enable us to determine the strengths and weaknesses of the NGSS--with the potential to identify conceptual gaps that ASHG might be able to address through its education activities--and may influence whether states adopt the NGSS as the basis for revising their curricula and assessments. Please note that in this analysis you are NOT evaluating the appropriateness of the core concepts on ASHG's list. Although that list is dynamic and will be updated periodically, for consistency we will use the same list as last time.

Appendix B - Summary of the Validation Process

We have limited to 28 the number of standards from the NGSS document that you need to examine. This was done to minimize the workload associated with reading large numbers of standards that are completely unrelated to genetics (i.e., the complete NGSS document). To identify those 28, we used a validation process involving seven genetics experts from the staff and Information and Education Committee. Of the 28 standards, 15 were independently and unanimously selected for inclusion and another three were selected by six experts. To make sure that we were not excluding any standards that could reasonably be viewed as genetics-related, we included 10 more standards that were identified by a minimum of two of the validation experts.
