# Supplementary material for: Assessing the Genetics Content in the Next Generation Science Standards
Source: PLoS One. 2015 Jul 29;10(7):e0132742. doi: 10.1371/journal.pone.0132742 (PMC4519196; doi:10.1371/journal.pone.0132742)
Supplement: S4 File — (PDF) [file pone.0132742.s004.pdf]

WIKIMEDIA COMMONS

Main page
Welcome
Community portal
Village pump
Help center

Language select
English
Select

Participate
Upload file
Recent changes
Latest files
Random file
Contact us

Tools
What links here
Related changes
Special pages
Permanent link
Page information
Nominate for deletion

File:Blank US map borders labels.svg

From Wikimedia Commons, the free media repository

File File history File usage on Commons File usage on other wikis

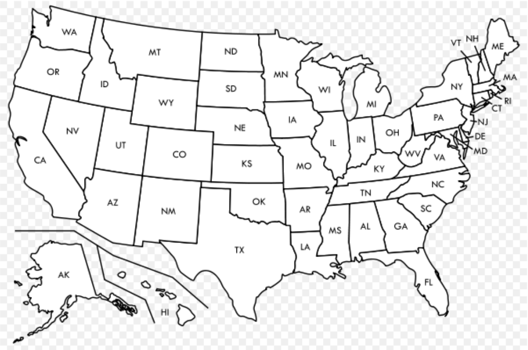

Download all sizes

Use this file on the web

Use this file on a wiki

Email a link to this file

Information about reusing

Size of this preview: 600 × 400 pixels. Other resolutions: 320 × 213 pixels | 640 × 427 pixels | 800 × 533 pixels | 1,024 × 683 pixels | 1,280 × 853 pixels.

Original file (SVG file, nominally 600 × 400 pixels, file size: 85 KB)

This image rendered as PNG in other widths: 200px, 500px, 1000px, 2000px.

Open in Media Viewer

Summary [edit]

**Description**	Labelled version of Image:Blank US map borders .svg
**Date**	28 October 2007
**Source**	self-made, based on CIA map found at http://www.lib.utexas.edu/maps/united_states/usa_ref01.pdf
**Author**	Kaboom88

Licensing [edit]

I, the copyright holder of this work, release this work into the **public domain**. This applies worldwide.

In some countries this may not be legally possible; if so:

*I grant anyone the right to use this work **for any purpose**, without any conditions, unless such conditions are required by law.*

File history

Click on a date/time to view the file as it appeared at that time.

	Date/Time	Thumbnail	Dimensions	User	Comment
current	**06:26, 29 October 2007**	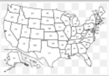	600 × 400 (85 KB)	Kaboom88~commonswiki (talk | contribs)	fixed hawaii and alaska
	06:03, 27 October 2007	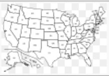	600 × 400 (83 KB)	Kaboom88~commonswiki (talk | contribs)	
	05:54, 27 October 2007	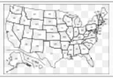	601 × 401 (82 KB)	Kaboom88~commonswiki (talk | contribs)	

- You cannot overwrite this file.

File usage on Commons

There are no pages that link to this file.

File usage on other wikis

The following other wikis use this file:

- Usage on en.wikipedia.org
  - User:Bettymnz4/Mid- to Late March 2008 southern Midwest floods

Category: Blank maps of the United States
Hidden category: PD-self

Text is available under the Creative Commons Attribution/Share-Alike License; additional terms may apply. By using this site, you agree to the Terms of Use and Privacy Policy.

Privacy policy About Wikimedia Commons Disclaimers Developers Mobile view
