# Supplementary material for: Assessing the Genetics Content in the Next Generation Science Standards
Source: PLoS One. 2015 Jul 29;10(7):e0132742. doi: 10.1371/journal.pone.0132742 (PMC4519196; doi:10.1371/journal.pone.0132742)
Supplement: S1 Table — Highlighted cells indicate “high-disagreement” concepts for each group. (PDF) [file pone.0132742.s005.pdf]

**S1 Table. Number of reviewers in the "NGSS only" and "NGSS+DCI" groups, including "outlier" reviewers who were later excluded, who assigned each score 0-2 for each concept.** Highlighted cells indicate "high-disagreement" concepts for each group.

| | **NGSS** | | | | **NGSS+DCI** | | | |
|---|---|---|---|---|---|---|---|---|
| | # of all reviewers assigning each score | | | (# of reviewers assigning least popular score)/(sum of reviewers assigning two most popular scores) | # of all reviewers assigning each score | | | (# of reviewers assigning least popular score)/(sum of reviewers assigning two most popular scores) |
| Core Concept | 0 | 1 | 2 | | 0 | 1 | 2 | |
| **1** | 3 | 6 | 6 | 25 | 2 | 2 | 7 | 22.2 |
| **2** | 1 | 8 | 6 | 7.1 | 0 | 3 | 13 | 0.0 |
| **3** | 7 | 8 | 0 | 0 | 3 | 5 | 3 | 37.5 |
| **4** | 0 | 5 | 10 | 0 | 1 | 10 | 5 | 6.7 |
| **5** | 2 | 10 | 3 | 15.4 | 10 | 1 | 0 | 0.0 |
| **6** | 10 | 3 | 2 | 15.4 | 13 | 2 | 1 | 6.7 |
| **7** | 10 | 5 | 0 | 0 | 10 | 1 | 0 | 0.0 |
| **8** | 6 | 6 | 3 | 25 | 8 | 7 | 1 | 6.7 |
| **9** | 8 | 6 | 1 | 7.1 | 0 | 4 | 7 | 0.0 |
| **10** | 10 | 5 | 0 | 0 | 5 | 8 | 3 | 23.1 |
| **11** | 1 | 7 | 7 | 7.1 | 1 | 5 | 5 | 10.0 |
| **12** | 0 | 7 | 8 | 0 | 2 | 3 | 11 | 14.3 |
| **13** | 0 | 4 | 11 | 0.0 | 2 | 2 | 7 | 22.2 |
| **14** | 10 | 3 | 2 | 15.4 | 9 | 6 | 1 | 6.7 |
| **15** | 0 | 2 | 13 | 0.0 | 1 | 0 | 10 | 10.0 |
| **16** | 8 | 6 | 1 | 7.1 | 10 | 6 | 0 | 0.0 |
| **17** | 0 | 4 | 11 | 0.0 | 0 | 3 | 8 | 0.0 |
| **18** | 1 | 3 | 11 | 7.1 | 0 | 1 | 15 | 0.0 |
| **19** | 0 | 6 | 9 | 0.0 | 0 | 3 | 8 | 0.0 |
