# Supplementary material for: Assessing the Genetics Content in the Next Generation Science Standards
Source: PLoS One. 2015 Jul 29;10(7):e0132742. doi: 10.1371/journal.pone.0132742 (PMC4519196; doi:10.1371/journal.pone.0132742)
Supplement: S2 Table — Difference was calculated as the average for "NGSS only" minus the state standards average [8] for each concept. Cells shaded orange indicate instances where the average "NGSS only" score is better by 0.3 or more. Blue shaded cells indicate instances where the average for the state standards is better by 0.3 or more. (PDF) [file pone.0132742.s006.pdf]

Table 2 - Difference between "NGSS only" and state standards by concept number. Difference was calculated as the average for "NGSS only" minus the state standards average (Dougherty et al., 2011.) for each concept. Cells shaded orange indicate instances where the average "NGSS only" score is better by 0.3 or more. Blue shaded cells indicate instances where the average for the state standards is better by 0.3 or more.

Standards	#1	#2	#3	#4	#5	#6	#7	#8	#9	#10	#11	#12	#13	#14	#15	#16	#17	#18	#19
**AK**	-0.7	-0.2	0.3	0.9	-0.4	0.2	0.2	0.4	0.4	-0.1	1.2	1.5	1.9	0.2	1.8	0.4	0.9	-0.1	0.7
**AL**	-0.7	-0.5	-1.4	-0.4	-1.1	-0.1	0.2	-0.6	0.1	0.2	1.2	-0.2	-0.1	0.2	0.8	-0.9	0.9	-0.1	1.1
**AR**	0.0	0.5	-0.7	0.6	-1.1	-0.5	0.2	-1.3	0.4	0.2	1.2	0.2	0.9	-0.1	-0.2	0.1	0.2	-0.1	-0.6
**AZ**	-0.4	-0.2	-0.1	-0.1	-0.1	-0.1	-0.1	-1.0	-0.6	-0.1	0.5	0.8	0.6	-0.5	1.5	0.1	-0.1	-0.1	-0.3
**CA**	-0.5	0.0	-1.2	-0.4	-1.1	0.2	0.2	-1.3	-1.1	-1.3	0.4	0.2	0.1	-0.1	0.3	-0.1	0.1	-0.2	0.9
**CO**	0.0	0.5	0.6	0.3	0.6	-0.1	-0.1	-0.3	0.4	0.2	-0.1	0.8	0.9	0.2	1.8	-0.6	0.2	-0.1	0.7
**CT**	-0.4	-0.5	-0.7	0.3	-1.1	-0.1	-0.1	-1.0	-0.6	-0.8	0.2	0.2	-0.1	-0.1	0.5	-0.9	-0.1	-0.4	-0.3
**DC**	-0.7	-0.5	-0.1	0.3	-1.1	0.2	0.2	-1.3	-1.3	-1.8	1.2	0.2	0.2	-0.5	0.1	0.4	0.2	-0.4	-0.3
**DE**	-0.7	-0.5	-1.4	-0.4	-1.1	-0.1	0.2	-1.3	-1.3	-0.8	-0.1	-0.5	-0.1	-1.8	-0.2	-1.6	-0.1	-0.4	-0.6
**FL**	-0.5	0.2	-1.2	-0.4	-1.1	-0.8	-0.8	-0.6	0.1	-0.1	0.9	0.2	0.1	-1.3	-0.2	-1.1	0.1	-0.2	0.6
**GA**	0.0	0.5	-0.4	0.3	-0.8	0.2	0.2	-0.6	-0.6	0.2	0.5	-0.5	-0.1	0.2	0.8	0.1	1.2	0.3	0.7
**HI**	-0.4	-0.2	-0.4	0.6	-1.1	-0.1	-0.1	-1.3	0.1	0.2	0.5	0.2	0.9	-0.5	0.8	-0.6	0.2	0.3	0.4
**IA**	-0.7	-0.5	-1.1	-0.4	-1.1	-0.1	-0.1	0.0	-0.3	0.2	0.2	-0.2	0.2	-0.5	-0.2	-1.3	-0.1	-0.4	-0.6
**ID**	0.0	0.2	0.6	1.6	0.9	0.2	0.2	0.2	-0.9	-1.1	0.2	1.5	1.9	0.2	1.8	0.4	0.4	-0.2	0.1
**IL**	-0.5	-0.5	-1.4	-0.4	-0.9	-0.8	-1.3	-0.8	-0.6	-0.3	-0.3	0.0	0.1	-0.8	0.3	-1.4	-0.1	0.1	0.1
**IN**	-0.7	0.5	0.1	0.6	-1.1	0.2	0.2	-0.3	-1.6	-1.1	-0.1	-0.3	-0.1	-1.8	-0.2	-1.1	0.1	-0.4	0.4
**KS**	-0.7	-0.5	-1.4	0.3	-0.1	-1.1	-1.8	-0.6	-0.3	-0.8	-0.5	0.2	0.2	-1.8	-0.2	-0.3	-0.1	-0.4	-0.6
**KY**	-0.7	-0.5	-0.4	0.6	0.2	-0.1	-0.1	-0.6	-0.9	-0.8	-0.1	-0.5	-0.1	-1.1	0.5	-0.6	0.6	-0.4	-0.3
**LA**	0.3	-0.2	0.3	0.3	-0.4	0.2	0.2	0.4	0.4	0.2	1.2	1.5	1.9	0.2	1.8	0.4	0.6	0.3	0.1
**MA**	-0.2	0.2	0.1	0.3	-1.1	-0.1	-0.3	-1.3	0.4	0.2	1.2	0.2	1.1	-0.3	0.0	0.4	0.6	0.3	-0.1
**MD**	-0.2	0.5	-1.2	-0.4	-0.9	0.2	0.2	-1.1	0.1	0.2	0.4	0.0	0.1	-1.1	-0.2	-0.4	0.1	-0.2	0.4
**ME**	-0.4	0.2	0.3	0.6	0.2	0.2	0.2	-0.3	-0.9	-1.1	0.5	0.5	0.9	0.2	0.5	-0.6	1.6	0.9	0.7
**MI**	-0.7	-0.5	-1.1	-0.1	-1.1	-1.1	-1.1	-1.3	-1.3	-0.8	-0.1	-0.5	-0.1	-1.8	-0.2	-1.6	-0.1	-0.4	-0.6
**MN**	-0.7	-0.5	-1.4	0.3	-1.1	-0.1	-0.1	-1.3	-0.6	-0.1	0.9	0.2	-0.1	-0.8	-0.2	-0.6	-0.1	-0.4	0.1
**MO**	-0.7	-0.5	-0.1	0.3	-1.1	-0.1	-0.1	0.0	0.1	0.2	0.9	-0.5	-0.1	-0.8	0.8	-0.6	1.9	1.6	1.4
**MS**	-0.4	0.2	-0.7	0.3	-1.1	0.2	0.2	-1.3	0.1	0.2	1.2	0.2	0.6	-0.5	0.8	-0.6	0.6	0.3	1.1
**MT**	-0.4	-0.2	-1.4	-0.4	-1.1	0.2	0.2	-1.0	-0.6	0.2	1.2	-0.2	0.2	-0.5	0.8	0.4	0.2	-0.1	-0.6
**NC**	-0.2	0.2	-1.4	-0.2	-1.1	-1.3	-1.8	-1.3	-0.9	-1.3	-0.3	0.5	0.6	-0.8	1.0	-0.9	0.4	-0.2	-0.1
**ND**	0.0	-0.5	-0.1	0.3	-1.1	0.2	0.2	0.4	0.4	-0.1	1.2	0.5	0.9	0.2	1.5	0.1	1.6	-0.1	0.7
**NE**	-0.2	1.2	0.6	0.8	0.9	0.2	0.2	0.7	0.4	0.2	1.2	0.7	0.1	-1.8	-0.2	-0.4	-0.1	-0.4	0.4
**NH**	-0.4	0.5	0.3	0.6	-0.8	-0.1	-0.1	0.4	-0.3	-0.8	0.2	-0.2	0.2	-1.8	-0.2	-1.3	-0.1	-0.4	0.1
**NJ**	0.0	0.7	0.3	0.3	-0.1	0.2	0.2	0.2	0.1	0.2	0.7	0.0	0.6	-0.1	-0.2	-0.1	0.9	0.3	0.6
**NM**	-0.7	-0.5	-1.1	-0.4	-0.8	-0.1	-0.1	-1.0	-0.9	-1.1	0.9	-0.5	0.2	0.2	0.8	-0.3	0.2	-0.4	0.7
**NV**	-0.7	0.8	-0.1	0.3	-0.8	0.2	0.2	-1.0	-1.6	-0.1	0.9	0.5	0.9	-1.8	1.5	0.1	0.6	-0.1	0.4
**NY**	-0.7	-0.5	0.3	0.8	0.6	0.2	-0.3	-0.1	-1.6	-0.6	-0.1	-0.5	0.4	-1.8	0.8	-1.1	0.1	-0.4	-0.6
**OH**	-0.7	0.2	-1.4	0.6	-1.1	0.2	-0.1	0.4	-0.3	-0.8	0.5	-0.2	0.6	-1.8	0.8	0.4	0.2	-0.4	0.4
**OK**	-0.4	0.5	-0.7	0.3	-0.4	-0.5	-0.5	-0.6	-0.9	-0.5	-0.1	1.2	1.6	0.2	1.5	0.4	0.9	0.3	0.1
**OR**	0.6	0.2	0.3	0.6	0.2	0.2	0.2	-1.0	0.1	-0.1	0.5	1.5	1.6	0.2	1.8	0.4	0.6	-0.1	0.7
**PA**	-0.4	-0.5	-1.4	-0.4	-0.8	0.2	0.2	-1.3	0.4	-0.1	1.2	0.2	0.2	-0.1	1.5	0.1	0.6	-0.4	-0.6
**RI**	-0.7	-0.2	0.3	-0.4	-0.8	0.2	0.2	-1.3	0.4	-0.1	0.2	-0.2	-0.1	-0.8	0.8	-0.9	-0.1	-0.4	-0.3
**SC**	-0.4	-0.5	0.6	0.6	-1.1	0.2	0.2	-1.3	0.1	-0.1	0.5	0.5	0.9	-1.8	0.8	0.4	0.2	-0.1	0.4
**SD**	0.0	0.8	-0.4	-0.1	-1.1	0.2	0.2	-0.3	-0.3	0.2	0.2	0.2	0.2	-0.5	0.8	-1.3	0.6	-0.1	0.1
**TN**	-0.7	-0.5	-0.7	-0.4	-1.1	-1.5	-1.8	-1.3	-0.9	-0.8	0.2	0.2	-0.1	-0.8	0.5	-1.6	0.9	-0.4	0.1
**TX**	-0.7	0.2	-1.1	0.3	-0.1	-0.5	-0.5	-1.0	-0.9	-1.5	-0.1	0.2	0.6	-1.1	0.8	-0.9	0.2	-0.1	-0.6
**UT**	-0.7	0.5	0.3	-0.4	-1.1	0.2	0.2	-0.3	-0.3	0.2	1.2	0.5	-0.1	-0.5	1.5	-0.6	0.2	-0.4	0.1
**VA**	-0.7	-0.2	-1.4	-0.1	-1.1	-0.1	0.2	-1.3	-1.3	0.2	0.9	-0.2	0.2	-0.8	-0.2	0.4	-0.1	-0.4	-0.6
**VT**	-0.5	0.0	-0.2	0.1	0.1	0.2	0.2	-0.3	-1.6	-1.8	0.2	-0.5	-0.1	-1.6	0.3	-1.1	0.4	-0.2	-0.1
**WA**	-0.7	-0.5	-1.1	-0.4	-0.8	-0.5	-0.5	-1.3	-1.3	-1.8	-0.1	-0.2	-0.1	-1.1	-0.2	-0.6	-0.1	-0.4	0.4
**WI**	-0.7	-0.3	-0.4	0.6	0.1	0.2	0.2	-0.1	-0.6	-1.1	0.2	0.2	1.1	-1.8	0.8	-0.1	1.1	0.1	0.6
**WV**	0.6	0.2	-0.7	0.3	-1.1	0.2	0.2	-1.3	-0.6	0.2	1.2	0.2	-0.1	-0.1	1.5	0.4	0.6	0.6	0.7
**WY**	0.0	1.0	0.1	0.6	0.4	0.2	0.2	-0.1	-0.4	-0.3	0.4	0.7	1.1	-0.3	1.0	0.1	0.1	-0.2	-0.4
