# Supplementary material for: Assessing the Genetics Content in the Next Generation Science Standards
Source: PLoS One. 2015 Jul 29;10(7):e0132742. doi: 10.1371/journal.pone.0132742 (PMC4519196; doi:10.1371/journal.pone.0132742)
Supplement: S3 Table — Difference was calculated as the average for "NGSS+DCI" minus the state standards average [8] for each concept. Cells shaded purple indicate instances where the average "NGSS+DCI" score is better by 0.3 or more. Green shaded cells indicate instances where the average for the state standards is better by 0.3 or more. (PDF) [file pone.0132742.s007.pdf]

Table 3 - Difference between "NGSS+DCI" and state standards by concept number. Difference was calculated as the average for "NGSS+DCI" minus the state standards average (Dougherty et al., 2011.) for each concept. Cells shaded purple indicate instances where the average "NGSS+DCI" score is better by 0.3 or more. Green shaded cells indicate instances where the average for the state standards is better by 0.3 or more.

Standards	#1	#2	#3	#4	#5	#6	#7	#8	#9	#10	#11	#12	#13	#14	#15	#16	#17	#18	#19
AK	-0.4	0.1	0.7	0.6	-1.3	0.3	0.1	0.2	1.6	0.5	1.5	1.8	1.8	0.6	2	0.4	0.9	0.2	1.2
AL	-0.4	-0.2	-1	-0.7	-2	0	0.1	-0.8	1.3	0.8	1.5	0.1	-0.2	0.6	1	-0.9	0.9	0.2	1.6
AR	0.3	0.8	-0.3	0.3	-2	-0.4	0.1	-1.5	1.6	0.8	1.5	0.5	0.8	0.3	0	0.1	0.2	0.2	-0.1
AZ	-0.1	0.1	0.3	-0.4	-1	0	-0.2	-1.2	0.6	0.5	0.8	1.1	0.5	-0.1	1.7	0.1	-0.1	0.2	0.2
CA	-0.2	0.3	-0.8	-0.7	-2	0.3	0.1	-1.5	0.1	-0.7	0.7	0.5	0	0.3	0.5	-0.1	0.1	0.1	1.4
CO	0.3	0.8	1	0	-0.3	0	-0.2	-0.5	1.6	0.8	0.2	1.1	0.8	0.6	2	-0.6	0.2	0.2	1.2
CT	-0.1	-0.2	-0.3	0	-2	0	-0.2	-1.2	0.6	-0.2	0.5	0.5	-0.2	0.3	0.7	-0.9	-0.1	-0.1	0.2
DC	-0.4	-0.2	0.3	0	-2	0.3	0.1	-1.5	-0.1	-1.2	1.5	0.5	0.1	-0.1	0.3	0.4	0.2	-0.1	0.2
DE	-0.4	-0.2	-1	-0.7	-2	0	0.1	-1.5	-0.1	-0.2	0.2	-0.2	-0.2	-1.4	0	-1.6	-0.1	-0.1	-0.1
FL	-0.2	0.5	-0.8	-0.7	-2	-0.7	-0.9	-0.8	1.3	0.5	1.2	0.5	0	-0.9	0	-1.1	0.1	0.1	1.1
GA	0.3	0.8	0	0	-1.7	0.3	0.1	-0.8	0.6	0.8	0.8	-0.2	-0.2	0.6	1	0.1	1.2	0.6	1.2
HI	-0.1	0.1	0	0.3	-2	0	-0.2	-1.5	1.3	0.8	0.8	0.5	0.8	-0.1	1	-0.6	0.2	0.6	0.9
IA	-0.4	-0.2	-0.7	-0.7	-2	0	-0.2	-0.2	0.9	0.8	0.5	0.1	0.1	-0.1	0	-1.3	-0.1	-0.1	-0.1
ID	0.3	0.5	1	1.3	0	0.3	0.1	0	0.3	-0.5	0.5	1.8	1.8	0.6	2	0.4	0.4	0.1	0.6
IL	-0.2	-0.2	-1	-0.7	-1.8	-0.7	-1.4	-1	0.6	0.3	0	0.3	0	-0.4	0.5	-1.4	-0.1	0.4	0.6
IN	-0.4	0.8	0.5	0.3	-2	0.3	0.1	-0.5	-0.4	-0.5	0.2	0	-0.2	-1.4	0	-1.1	0.1	-0.1	0.9
KS	-0.4	-0.2	-1	0	-1	-1	-1.9	-0.8	0.9	-0.2	-0.2	0.5	0.1	-1.4	0	-0.3	-0.1	-0.1	-0.1
KY	-0.4	-0.2	0	0.3	-0.7	0	-0.2	-0.8	0.3	-0.2	0.2	-0.2	-0.2	-0.7	0.7	-0.6	0.6	-0.1	0.2
LA	0.6	0.1	0.7	0	-1.3	0.3	0.1	0.2	1.6	0.8	1.5	1.8	1.8	0.6	2	0.4	0.6	0.6	0.6
MA	0.1	0.5	0.5	0	-2	0	-0.4	-1.5	1.6	0.8	1.5	0.5	1	0.1	0.2	0.4	0.6	0.6	0.4
MD	0.1	0.8	-0.8	-0.7	-1.8	0.3	0.1	-1.3	1.3	0.8	0.7	0.3	0	-0.7	0	-0.4	0.1	0.1	0.9
ME	-0.1	0.5	0.7	0.3	-0.7	0.3	0.1	-0.5	0.3	-0.5	0.8	0.8	0.8	0.6	0.7	-0.6	1.6	1.2	1.2
MI	-0.4	-0.2	-0.7	-0.4	-2	-1	-1.2	-1.5	-0.1	-0.2	0.2	-0.2	-0.2	-1.4	0	-1.6	-0.1	-0.1	-0.1
MN	-0.4	-0.2	-1	0	-2	0	-0.2	-1.5	0.6	0.5	1.2	0.5	-0.2	-0.4	0	-0.6	-0.1	-0.1	0.6
MO	-0.4	-0.2	0.3	0	-2	0	-0.2	-0.2	1.3	0.8	1.2	-0.2	-0.2	-0.4	1	-0.6	1.9	1.9	1.9
MS	-0.1	0.5	-0.3	0	-2	0.3	0.1	-1.5	1.3	0.8	1.5	0.5	0.5	-0.1	1	-0.6	0.6	0.6	1.6
MT	-0.1	0.1	-1	-0.7	-2	0.3	0.1	-1.2	0.6	0.8	1.5	0.1	0.1	-0.1	1	0.4	0.2	0.2	-0.1
NC	0.1	0.5	-1	-0.5	-2	-1.2	-1.9	-1.5	0.3	-0.7	0	0.8	0.5	-0.4	1.2	-0.9	0.4	0.1	0.4
ND	0.3	-0.2	0.3	0	-2	0.3	0.1	0.2	1.6	0.5	1.5	0.8	0.8	0.6	1.7	0.1	1.6	0.2	1.2
NE	0.1	1.5	1	0.5	0	0.3	0.1	0.5	1.6	0.8	1.5	1	0	-1.4	0	-0.4	-0.1	-0.1	0.9
NH	-0.1	0.8	0.7	0.3	-1.7	0	-0.2	0.2	0.9	-0.2	0.5	0.1	0.1	-1.4	0	-1.3	-0.1	-0.1	0.6
NJ	0.3	1	0.7	0	-1	0.3	0.1	0	1.3	0.8	1	0.3	0.5	0.3	0	-0.1	0.9	0.6	1.1
NM	-0.4	-0.2	-0.7	-0.7	-1.7	0	-0.2	-1.2	0.3	-0.5	1.2	-0.2	0.1	0.6	1	-0.3	0.2	-0.1	1.2
NV	-0.4	1.1	0.3	0	-1.7	0.3	0.1	-1.2	-0.4	0.5	1.2	0.8	0.8	-1.4	1.7	0.1	0.6	0.2	0.9
NY	-0.4	-0.2	0.7	0.5	-0.3	0.3	-0.4	-0.3	-0.4	0	0.2	-0.2	0.3	-1.4	1	-1.1	0.1	-0.1	-0.1
OH	-0.4	0.5	-1	0.3	-2	0.3	-0.2	0.2	0.9	-0.2	0.8	0.1	0.5	-1.4	1	0.4	0.2	-0.1	0.9
OK	-0.1	0.8	-0.3	0	-1.3	-0.4	-0.6	-0.8	0.3	0.1	0.2	1.5	1.5	0.6	1.7	0.4	0.9	0.6	0.6
OR	0.9	0.5	0.7	0.3	-0.7	0.3	0.1	-1.2	1.3	0.5	0.8	1.8	1.5	0.6	2	0.4	0.6	0.2	1.2
PA	-0.1	-0.2	-1	-0.7	-1.7	0.3	0.1	-1.5	1.6	0.5	1.5	0.5	0.1	0.3	1.7	0.1	0.6	-0.1	-0.1
RI	-0.4	0.1	0.7	-0.7	-1.7	0.3	0.1	-1.5	1.6	0.5	0.5	0.1	-0.2	-0.4	1	-0.9	-0.1	-0.1	0.2
SC	-0.1	-0.2	1	0.3	-2	0.3	0.1	-1.5	1.3	0.5	0.8	0.8	0.8	-1.4	1	0.4	0.2	0.2	0.9
SD	0.3	1.1	0	-0.4	-2	0.3	0.1	-0.5	0.9	0.8	0.5	0.5	0.1	-0.1	1	-1.3	0.6	0.2	0.6
TN	-0.4	-0.2	-0.3	-0.7	-2	-1.4	-1.9	-1.5	0.3	-0.2	0.5	0.5	-0.2	-0.4	0.7	-1.6	0.9	-0.1	0.6
TX	-0.4	0.5	-0.7	0	-1	-0.4	-0.6	-1.2	0.3	-0.9	0.2	0.5	0.5	-0.7	1	-0.9	0.2	0.2	-0.1
UT	-0.4	0.8	0.7	-0.7	-2	0.3	0.1	-0.5	0.9	0.8	1.5	0.8	-0.2	-0.1	1.7	-0.6	0.2	-0.1	0.6
VA	-0.4	0.1	-1	-0.4	-2	0	0.1	-1.5	-0.1	0.8	1.2	0.1	0.1	-0.4	0	0.4	-0.1	-0.1	-0.1
VT	-0.2	0.3	0.2	-0.2	-0.8	0.3	0.1	-0.5	-0.4	-1.2	0.5	-0.2	-0.2	-1.2	0.5	-1.1	0.4	0.1	0.4
WA	-0.4	-0.2	-0.7	-0.7	-1.7	-0.4	-0.6	-1.5	-0.1	-1.2	0.2	0.1	-0.2	-0.7	0	-0.6	-0.1	-0.1	0.9
WI	-0.4	0	0	0.3	-0.8	0.3	0.1	-0.3	0.6	-0.5	0.5	0.5	1	-1.4	1	-0.1	1.1	0.4	1.1
WV	0.9	0.5	-0.3	0	-2	0.3	0.1	-1.5	0.6	0.8	1.5	0.5	-0.2	0.3	1.7	0.4	0.6	0.9	1.2
WY	0.3	1.3	0.5	0.3	-0.5	0.3	0.1	-0.3	0.8	0.3	0.7	1	1	0.1	1.2	0.1	0.1	0.1	0.1
