# Supplementary material for: Assessing the Genetics Content in the Next Generation Science Standards
Source: PLoS One. 2015 Jul 29;10(7):e0132742. doi: 10.1371/journal.pone.0132742 (PMC4519196; doi:10.1371/journal.pone.0132742)
Supplement: S4 Table — Highlighted cells indicate “unanimous/nearly unanimous” core concepts. (PDF) [file pone.0132742.s008.pdf]

S4 Table. Number of included reviewers in the "NGSS only" and "NGSS+DCI" groups (excluding "outlier" reviewers) who assigned each score 0-2 for each concept. Highlighted cells indicate "unanimous/nearly unanimous" core concepts.

	**NGSS**				**NGSS+DCI**			
	# of included reviewers assigning each score			(# of reviewers assigning least popular score)/(sum of reviewers assigning two most popular scores)	# of included reviewers assigning each score			(# of reviewers assigning least popular score)/(sum of reviewers assigning two most popular scores)
Core Concept	0	1	2		0	1	2	
**1**	1	4	4	12.5%	1	1	6	14.3%
**2**	0	7	6	0.0%	0	2	9	0.0%
**3**	4	5	0	0.0%	2	4	2	33.3%
**4**	0	5	8	0.0%	0	8	3	0.0%
**5**	1	8	0	0.0%	8	0	0	0.0%
**6**	11	1	1	12.5%	9	1	1	10.0%
**7**	7	2	0	0.0%	7	1	0	0.0%
**8**	6	5	2	22.2%	5	6	0	0.0%
**9**	5	4	0	0.0%	0	3	5	0.0%
**10**	10	3	0	0.0%	4	5	2	22.2%
**11**	1	5	3	12.5%	0	4	4	0.0%
**12**	0	6	7	0.0%	0	2	9	0.0%
**13**	0	1	8	0.0%	0	2	6	0.0%
**14**	11	2	0	0.0%	5	5	1	10.0%
**15**	0	2	7	0.0%	0	0	8	0.0%
**16**	8	5	0	0.0%	7	4	0	0.0%
**17**	0	1	8	0.0%	0	1	7	0.0%
**18**	1	3	9	12.5%	0	1	10	0.0%
**19**	0	5	4	0.0%	0	1	7	0.0%
